# Novel calpain families and novel mechanisms for calpain regulation in *Aplysia*

**DOI:** 10.1371/journal.pone.0186646

**Published:** 2017-10-20

**Authors:** Margaret H. Hastings, Katrina Gong, Alexander Freibauer, Caitlin Courchesne, Xiaotang Fan, Wayne S. Sossin

**Affiliations:** 1 Department of Psychology Montreal Neurological Institute McGill University, Montreal, Quebec, Canada; 2 Department of Neurology and Neurosurgery, Montreal Neurological Institute, McGill University, Montreal, Quebec, Canada; Russian Academy of Medical Sciences, RUSSIAN FEDERATION

## Abstract

Calpains are a family of intracellular proteases defined by a conserved protease domain. In the marine mollusk *Aplysia californica*, calpains are important for the induction of long-term synaptic plasticity and memory, at least in part by cleaving protein kinase Cs (PKCs) into constitutively active kinases, termed protein kinase Ms (PKMs). We identify 14 genes encoding calpains in *Aplysia* using bioinformatics, including at least one member of each of the four major calpain families into which metazoan calpains are generally classified, as well as additional truncated and atypical calpains. Six classical calpains containing a penta-EF-hand (PEF) domain are present in *Aplysia*. Phylogenetic analysis determined that these six calpains come from three separate classical calpain families. One of the classical calpains in *Aplysia*, AplCCal1, has been implicated in plasticity. We identify three splice cassettes and an alternative transcriptional start site in AplCCal1. We characterize several of the possible isoforms of AplCCal1 *in vitro*, and demonstrate that AplCCal1 can cleave PKCs into PKMs in a calcium-dependent manner *in vitro*. We also find that AplCCal1 has a novel mechanism of auto-inactivation through N-terminal cleavage that is modulated through its alternative transcriptional start site.

## Introduction

Calpains are an ancient and highly conserved superfamily of intracellular proteases with diverse roles in cellular physiology. Homologs, defined by the protease domain which is distantly related to papains, have been found in members of all six major supergroups into which eukaryotes are currently classified [1] and in bacteria [2]. Calpains are structurally diverse and many eukaryotes possess multiple distinct calpain isoforms [1]. Within metazoa, 4 distinct calpain families are recognized based on structural differences: Classical, Small Optic Lobe (SOL), Transformer 3 (Tra) and PalB (named after the screen for acid-sensitive phosphatase mutants in the fungi, Aspergillus) families. Calpains are implicated in fundamental cellular processes including apoptosis [3] and cell division [4] and overactivation or loss of calpains is implicated in a number of pathologies [5–8].

In the nervous system, calpains have important roles in initiating synaptic plasticity. Calpain inhibition disrupts plasticity and memory in both vertebrate [9–13] and invertebrate models [14–18]. Most work on vertebrate plasticity has focused on two members of the Classical, penta-EF-hand (PEF) domain-containing calpain family, Calpain-1 (CAPN-1) and Calpain-2 (CAPN-2), which are calcium (Ca^2+^)-dependent proteases that may also be activated by phosphorylation [19, 20]. CAPN-1 is required for theta-burst stimulation (TBS)-induced long-term synaptic potentiation (LTP) and memory [20, 21] in which it acts partly through degradation of Suprachiasmatic Nucleus Circadian Oscillatory Protein (SCOP) to activate MAP kinase [12, 21, 22]. CAPN-1 has also recently been implicated in mGluR-dependent long-term depression (LTD) and memory extinction, where it acts partly through cleavage of PP2A subunit B56α [13]. CAPN-2 has an enigmatic role in LTP, as loss of CAPN-2 activity has been reported either to disrupt [23] or enhance [21, 22] LTP and memory. The complexity of the calpain system in plasticity is further illustrated by the finding that inhibition of CAPN-2 rescues TBS-LTP and memory in CAPN-1 knockout mice [24]. Calpain-dependent cleavage has been demonstrated for a number of different synaptic proteins, consistent with the idea that calpains play many roles in mammalian plasticity [25–29].

Calpains have long been known to cleave Protein Kinase C (PKC) to form a constitutively active kinase called protein kinase M (PKM) [30–32]. Most work on PKM-dependent memory and plasticity has focused on the rodent PKM zeta isoform [33], which is formed through a non-calpain-dependent mechanism; i.e., translation from an alternative transcript [34]. However, the alternative transcript is present only in vertebrates [35] and evidence points to calpain-mediated PKM formation as a critical mechanism for memory and plasticity in invertebrates, suggesting this may be the ancestral mechanism for PKM formation in memory [14–18, 36, 37].

The strongest evidence for calpain-mediated PKM formation is in the synaptic plasticity underlying sensitization of the defensive withdrawal reflex in the mollusk *Aplysia californica*, a model system that permits overexpression of recombinant proteins in the pre- or post-synaptic cell in cultures that recapitulate the plasticity that occurs in the animal during memory [38]. Despite the absence of alternative transcripts encoding PKM in *Aplysia* [35, 39], the pharmacological inhibitors zeta-inhibitory peptide (ZIP) and chelerythrine, which are effective against all PKM forms of the *Aplysia* PKCs [17], disrupt the maintenance of both long-term sensitization and long-term synaptic facilitation (LTF) [40, 41]. Chelerythrine also disrupted the maintenance of site-specific sensitization [14] and memory that food is inedible [42]. Moreover, FRET-based cleavage reporter constructs generated from the *Aplysia* classical PKC Apl I and the atypical PKC Apl III undergo cleavage after induction of distinct forms of synaptic plasticity in sensory-motor neuron cultures [17, 36]. This plasticity-related PKC cleavage is mediated by calpain, as it could be blocked with a calpain inhibitor or by overexpression of a dominant negative form of the *Aplysia* classical calpain AplCCal1 [17]. Dominant negative AplCCal1 also blocked induction of three forms of synaptic plasticity modeling different forms of sensitization [17, 18, 43]. Interestingly, a recent finding suggests a role for a non-classical calpain in synaptic plasticity in *Aplysia* as well. A dominant-negative form of the *Aplysia* small optic lobes (SOL) calpain (AplSOL) impaired induction of non-associative LTF [18, 43].

Despite the evidence for a role for calpains in plasticity in *Aplysia*, there is currently no information on the total number of *Aplysia* calpains and their relationships to the better-characterized mammalian calpains implicated in plasticity. Furthermore, the activity of *Aplysia* calpains, including the calpain most strongly implicated in plasticity, AplCCal1, has not been confirmed or characterized *in vitro*. Here we define the *Aplysia* calpain family and its relationship to other calpains. Through this effort, we also have discovered new calpain families and better defined the evolutionary history of calpains. We also characterize AplCCal1 catalytic activity, identifying a mechanism of autoinactivation by N-terminal cleavage not previously observed in the classical calpain family.

## Methods

### Phylogenetic analysis

We selected species to sample a range of bilaterian and pre-bilaterian branches. We included additional members of Lophotrochozoa to better define the *Aplysia* calpains as *Aplysia* is a member of this class. All organisms are listed in Table 1 and the phylogenetic relationship of these animals is described in S1 Fig.

**Table 1:** Organisms used in Phylogenetic analysis.

Classification	Scientific Name	Abbreviation	Common Name	Comments
Deuterostomes	Strongylocentrotus Purpuratus	PUR	Sea Urchin	
	Branchiostoma Floridae	BRA	Tunicate	
	Danio Rerio	DAN	Zebrafish	
	Homo Sapiens	HUM	Human	
	Xenopus Tropicale	XEN	Frog	
	Fugu Rubripes	FUG	Pufferfish	Only CAPN17
Ecdysozoa	Drosophila Melanogaster	DRO	Fruit Fly	
	Daphnia Pulex	DAP	Water flea	
	Limulus polyphemus	LIM	Horseshoe Crab	Only if not in DRO or DAP
Lophotrochozoa	Aplysia californica	APL	Aplysia	
	Lottia Gigantica	LOT	Limpet	
	Crassostrea Gigas	CRA	Oyster	
	Capitella teleta	CAP	Polychaete Worm	
Pre-bilaterian	Amphimedon Queenslandica	AMP	Sponge	
	Trichoplax Adhaerens	TRI	Placozoa	
	Nematostella Vectensis	NEM	Sea Anemone	
	Mnemiopsis Leidyi	MNE	Comb Jelly	
Pre-metazoan	Capsaspora Owczarzaki	CAS	Filasterean	
	Salpingoeca Rosetta	SAL	Choanoflagellate	
	Histoplasma	HIS	Fungi	

List of Species used in phylogenetic analysis

Amino acid sequences were drawn from the NCBI Protein database, the *Aplysia* transcriptome database at https://Aplysiagenetools, and the *Mnemiopsis* database at https://research.nhgri.nih.gov/mnemiopsis, using BLAST searches (See S1 Table for accession numbers). For AplSOL and AplCCal1 we used the sequence of our own clones. Reverse BLAST searches were done to ensure that only true calpain homologs were included in the phylogeny. Thus, for a sequence to be identified as a calpain, the closest relatives identified by BLAST search must be calpains. We also excluded several calpains because of their strong divergence in the catalytic domain that made phylogenetic analysis difficult. This included two calpains from Capitella (Accession numbers ELU17011 and ELU07534.1), one from Mnenopsis (ML070242 (Mnemiopsis leidyi prot2.2.aa.fa), one from Nematostella (XP_001640599.1). Evolutionary analysis was performed similar to previous reports [39, 44]. For the analysis over all calpain family members, we used a highly conserved region of approximately 160–170 amino acids within the catalytic domain, beginning at the start of the Cys/PC domain and ending at the sequence TGX. For the analysis limited to classical calpain catalytic domains, this region was extended to the entire PFAM Cys/PC domain of about 300 amino acids. Both Phylip and Randomized Axelerated Maximum Likelihood (RAxML) programs [45, 46] were used for phylogenetic analysis. For Phylip, Sequences were aligned with Clustal-W, 1000 replicates were generated with the Phylip program Seqboot, and then the Phylip program ProtDist was used with the Jones-Taylor-Thornton model to generate a Distance Matrix. The Phylip program Neighbor was used to generate trees from each repetition, the program Consense used to generate the consensus tree and Drawgram used to make the final tree shown. For RAxML, the same clustalW file was subjected to ML+rapid bootstrap with 1000 reps and PROTCATI used for the substitute model and LG used for the substitution matrix. A plant calpain sequence, Rice DEK, was used as an outgroup. The RaXML trees are shown and differences with Phylip neighbor-joining trees are discussed in the text. Previously unnamed calpains that fell within a strong clade (bootstrap value > 95%) were named after that clade (ie. SOL, PalB, Atypical). Calpains that were not associated with a strong clade were named based on the domain structure of the predicted protein sequence, with CCAL signifying the presence of an EF-hand domain, TRA signifying a C2 domain, TRUNC signifying an isolated catalytic and C2-like (C2L) domain, and CAT signifying an isolated catalytic domain. Note that some truncated protein sequences may reflect incomplete transcriptome assemblies.

### Constructs

Cloning and baculovirus production for AplCCal1, 1a and 1b, PKC Apl I, PKC Apl III has been described [35, 47, 48]. The CCal1alt unique N-terminal portion, along with a stretch of adjacent sequence common to both alternative transcripts was amplified from *Aplysia* nervous system cDNA (RNAqueous total RNA isolation kit and Superscript II reverse transcriptase, Thermo Fisher Scientific) using PCR with nested primers (5’ outer: GGAAGCTAGCAGGCATTCC, 5’ inner: GAGCTCCCATGTCTAACTACTACAAGACCC; 3’ outer: ATCACTCCAAGCACCTGTCC, 3’ inner: CCATAATGAGTCCGTTGGCC), cloned into pJET1.2 vector using the CloneJET PCR cloning kit (Thermo-Scientific) and used to replace the N-terminal region of AplCCal1 in pFastBac HT-A vector through digestion of both plasmids with SacI and ClaI followed by ligation. The C-S mutant was previously generated [17] in the pNEX3 vector and was transferred to the baculovirus vector using appropriate restriction enzymes. Baculovirus was generated in *Spodoptera frugiperda* (Sf9) cells using the Bac-to-Bac baculovirus expression system according to the manufacturer’s instructions (Life Technologies Inc, Burlington, Ontario).

### Expression and purification of recombinant proteins

Sf9 cells in suspension (100ml) were infected with baculovirus. Three days after infection, His-tagged calpain or PKC was purified using Pro-bond His-Affinity resin (Life Technologies Inc), in modified purification buffer (20mM HEPES pH 7.5, 1mM DTT, 100mM KCl, 10% glycerol, with 1mM EDTA added for calpain purifications or 10mM MgCl_2_ for PKC or PKM). Proteins were bound to 1ml of beads in purification buffer containing 10mM imidazole, washed four times with purification buffer containing 20mM imidazole, and then eluted in purification buffer with 0.25M imidazole, 1ml at a time for a total of 4ml. DTT was added to a final concentration of 11mM, and the sample was concentrated using Amicon Ultra-15 Centrifugal Filter Units (Millipore Sigma) and stored at -80°C.

### Antibodies

Generation and characterization of the polyclonal rabbit antibodies used were previously described: PKC Apl III [36]; PKC Apl I [47]; and AplCCal1 C-termini [17].

### Calpain assays

Purified recombinant *Aplysia* calpain or CAPN-1 from porcine erythrocytes (Calbiochem Cat. No 208712) was incubated alone or with purified recombinant PKC Apl I, III or casein (Hammarsten bovine, Sigma-Aldrich, E0789, 5mg/ml stock in 0.75M Tris, pH8) for the times indicated, in activation buffer (5mM L-cysteine, 100mM Imidazole, 5mM CaCl_2_ unless otherwise indicated) or no- CaCl_2_ vehicle, at 30°C for porcine calpain unless otherwise indicated, at room temperature for *Aplysia* calpains. In experiments testing the effect of calpain inhibitors, calpain and casein were preincubated with 100 uM PD150606 (Santa Cruz Biotechnology, stock: 50mM in DMSO) or 100 uM ALLM (Santa Cruz Biotechnology, stock: 50mM in DMSO) or vehicle for 20min, followed by addition of activation buffer with 100uM PD150606, ALLM or vehicle. In Ca^2+^ preincubation experiments, calpain was preincubated with 5mM CaCl_2_ activation buffer or vehicle for 30min prior to addition of casein along with 5mM CaCl_2_ activation buffer or vehicle. As porcine CAPN-1 was supplied in a different buffer (20mM imidazole, 5mM beta-mercaptoethanol, 1mM EDTA, 1mM EGTA, 30% glycerol) from the AplCCal1 elution buffer, assays directly comparing the activity of these two calpains against casein were modified to keep the reaction buffers identical between groups.

### Animals

*Aplysia californica* were obtained from the University of Miami *Aplysia* Resource Facility (RSMAS, Miami, FL) and maintained in a saltwater aquarium.

### Treatment and homogenization of ganglia

For ionomycin treatment, pleuropedal ganglia were stored after dissection at 4°C for up to 2 days, digested with Dispase II (Roche) at 10mg/mL L15 for 1h and 50min at 37°C, desheathed and treated with 100uM ionomycin in ASW with 5% DMSO or vehicle 20min at RT and immediately homogenized with a disposable tissue grinder in ice-cold homogenization buffer (20mM HEPES pH 7.5, 0.5mM EDTA, 0.5mM EGTA, 2.6mM 2-mercaptoethanol, 50mM NaF, 5mM Sodium Pyrophosphate, 10% glycerol, Roche Complete EDTA-free protease inhibitor cocktail). For visualization of endogenous PKC/PKM, ganglia were incubated at 19°C for 2h after dissection and then homogenized without Dispase treatment or desheathing. After a 30s centrifugation at 13000 rpm, the supernatant was collected and protein concentration was determined by Bradford assay. Proteins were denatured by addition of Laemmli sample buffer and heating at 95°C before being subjected to SDS-PAGE and immunoblotting. Ten to 20μg of total protein were loaded.

### Statistics

One-tailed t-tests were used in all analysis as the direction of anticipated effects were known. Paired t-tests were used for data comparing the effect of two treatments on the two paired pleural-pedal ganglia from the same animal, or on two samples from a single batch of purified protein. When the effect of a treatment on two different purified proteins were compared, a t-test for independent samples was used.

## Results

### Defining the *Aplysia* calpain family

Our search for *Aplysia* calpains yielded 14 unique proteins with a full catalytic domain. The search also yielded a protein with an incomplete catalytic domain, which we identified as a homolog of the vertebrate protein androglobin, previously known as CAPN16 or demi-calpain before its recognition as a member of the globin superfamily [49]. Androglobins were not included in any of the analyses that follow.

We first attempted to fit the *Aplysia* calpains into known families based on their structure. The calpains present in animals are classified into four conserved families defined by domains outside the catalytic domain: SOL defined by N-terminal zinc fingers and a SOL domain replacing the C2-like domain III (C2L) present in all other calpain families, TRA defined by a C-terminal C2 domain, PalB defined by an N-terminal MIT domain and duplicate C2L domains, and classical calpains, defined by a C-terminal PEF domain [50]. Based on the presence of these conserved regions, 1 SOL calpain (AplSOL), 1 PalB calpain (AplPalB), 2 TRA calpains (AplTra1, AplTra2) and 6 classical calpains (AplCCal1-6) are present in *Aplysia*. There were 4 additional calpains that did not contain any of these identifying sequences. One, which we are calling atypical calpain (AplAty), has two C2L domains, a structure similar to CAPN-10 in vertebrates. The remaining three are truncated, consisting only of the catalytic domain with an adjoining C2L domain (AplTrunc1-3).

#### Relationship of *Aplysia* calpains to calpains in other species

To determine the relationship between the *Aplysia* calpains and those in other species, we performed a phylogenetic analysis with all calpains we could obtain from species belonging to a range of key phyla for which extensive transcript and predicted protein sequence information is available. These species included members of the deuterostomia, lophotrochozoa, ecdysozoa, prebilaterian metazoans, several sister groups of metazoa, and fungi (Table 1; S1 Fig). Note that the numbering of *Aplysia* and other non-vertebrate calpains is arbitrary and unrelated to the established numbering of vertebrate isoforms. We focused on a highly conserved region in the catalytic domain (see Methods) using methods similar to previous examination of the phylogenetic relationships of invertebrate receptor tyrosine kinases, phospholipases and PKCs [39, 44, 51].

**Fig 1:**
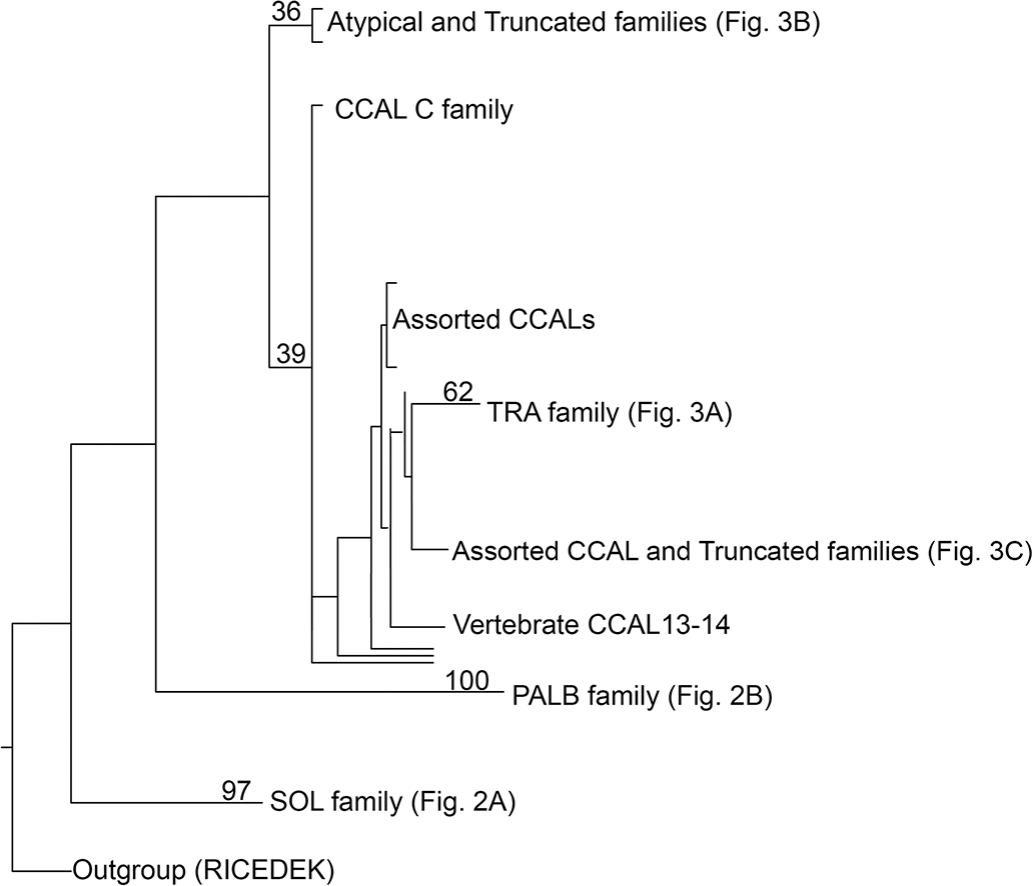
Phylogeny of calpain families. An overview of the analysis of all Calpain families is presented. The analysis is described in the methods (the plot is from the RAxML analysis). Numbers represent the percentage of trees containing this phylogeny. Regions of the tree are expanded in the figures noted. The vertebrate CCAL 13–14 family, CCAL C family and assorted CCALs are examined more fully later in the paper. The full tree can be seen in S2 Fig.

**Fig 2:**
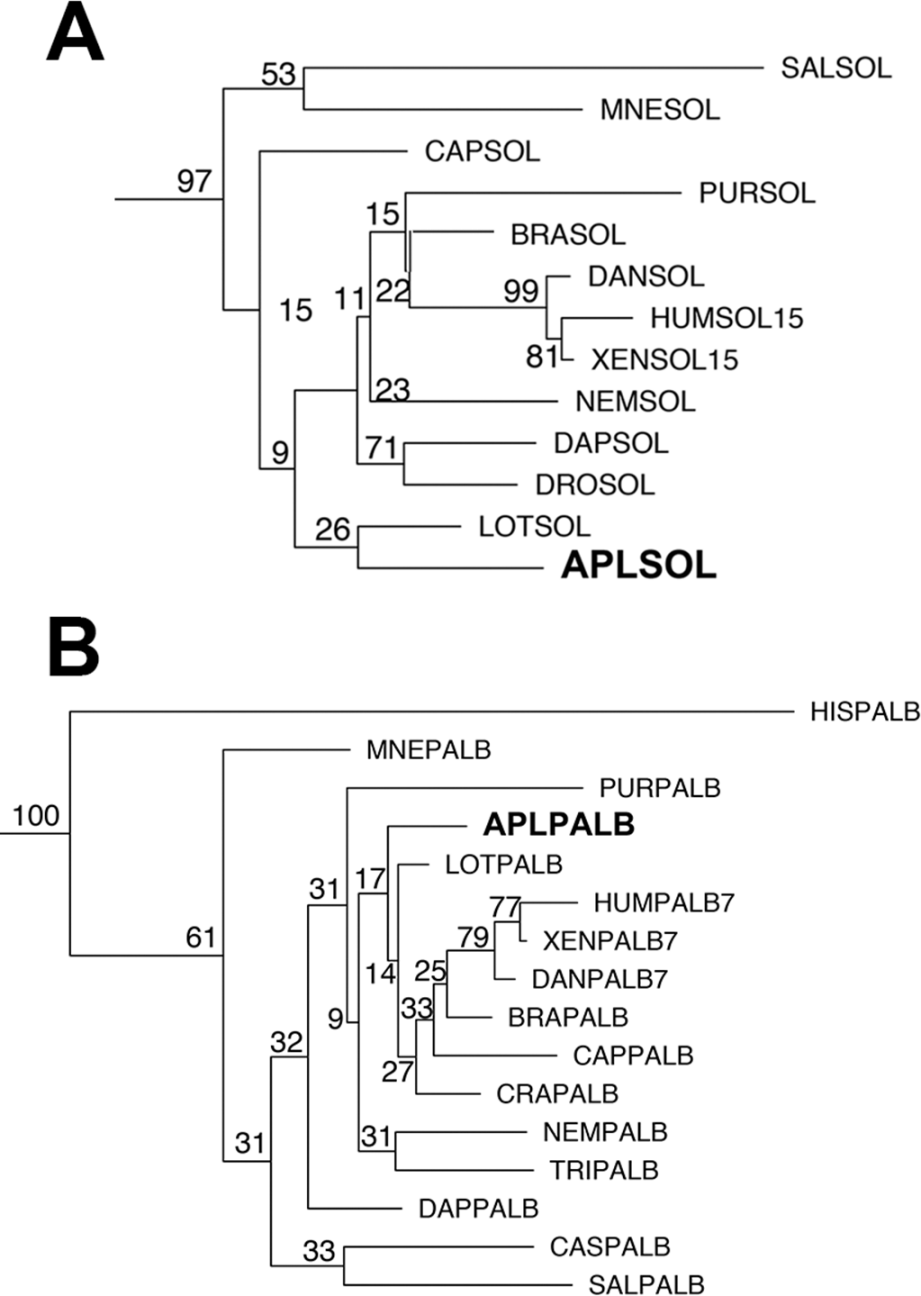
Phylogeny of SOL and PalB calpain families. Species abbreviations and their phylogenetic classification and common name are detailed in Table 1. All reference numbers for sequences are in S1 Table. The analysis is described in the methods (the plot is from the RAxML analysis). *Aplysia* calpains are in larger font. (A) SOL calpain family. (B) PalB calpain family.

With the large number of sequences (128) used in this analysis, it is difficult to show all details on one figure. We show the general outline of the results in Fig 1 and the complete large figure in S2 Fig. We expand selected regions as described below. Our phylogeny (Fig 1) is consistent with preexisting reports that SOL and PalB represent ancient, well-defined calpain clades [2]. The conserved region of the catalytic domain is sufficient to group calpains from these clades, drawn from metazoa and choanoflagellates (*Salpingoeca*; sister group of metazoa), and, in the case of PalB, filasterea (*Capsaspora;* sister group of metazoa) and fungi (*Histoplasma)*, with high confidence (Fig 2). The phylogeny confirms the placement of the *Aplysia* SOL and PalB calpains, which we originally classified based on domain structure alone, in their respective families (Fig 2).

**Fig 3:**
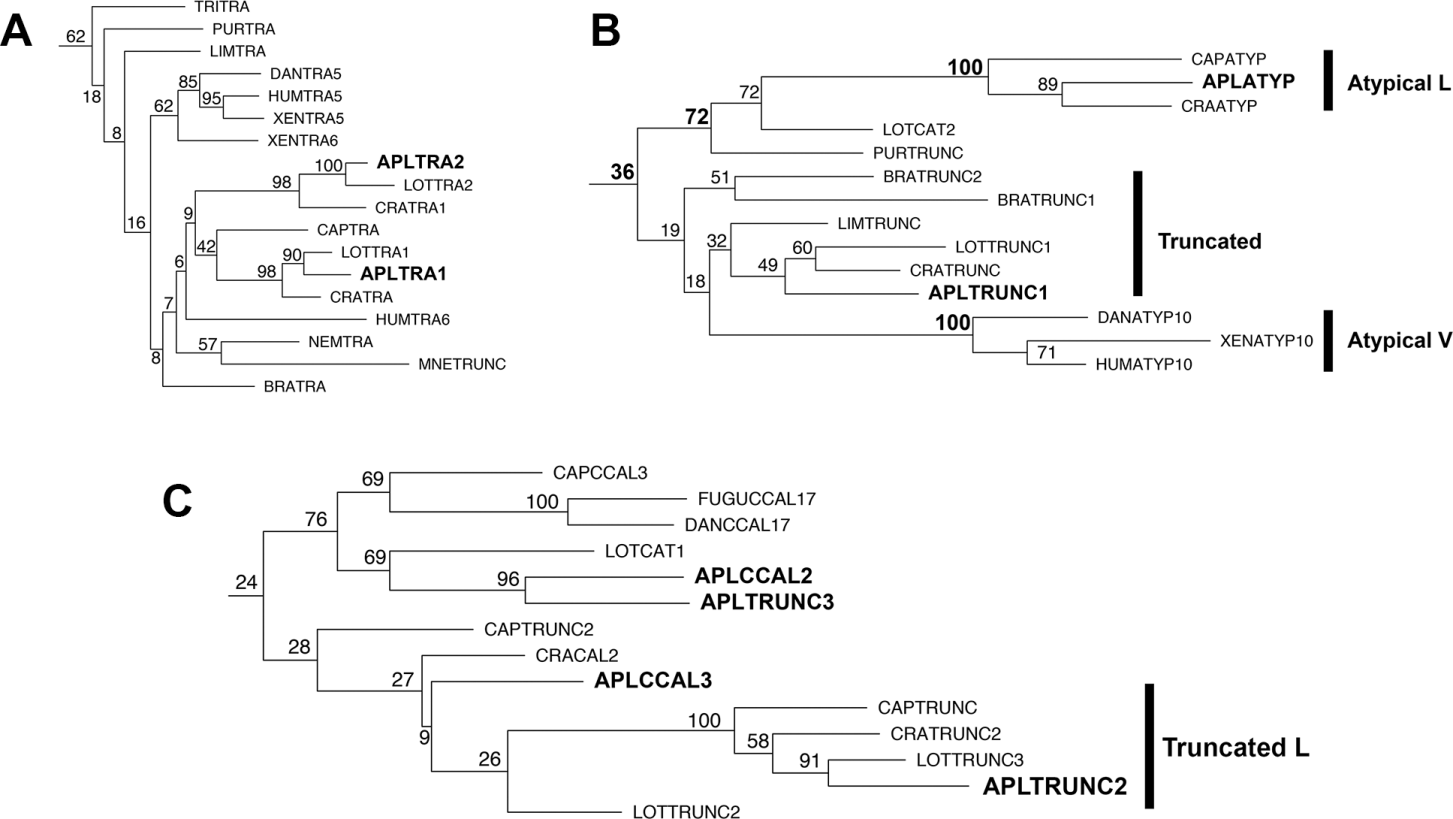
Phylogeny of Tra, Atypical and Truncated families. Species abbreviations and their phylogenetic classification and common name are detailed in Table 1. All reference numbers for sequences are in S1 Table. The analysis is described in the methods (the plot is from the RAxML analysis). *Aplysia* calpains are in larger font and bolded as are bootstrap numbers referred to in the text that define families. When multiple families are present, the families are defined by the lines and the family name on the right. (A) Tra family, (B) Atypical and Truncated families (C) Truncated family L and associated Truncated and Classical calpains.

There was a clear clade consisting of the Tra family, albeit with lower bootstrap values (62) than the SOL or Palb families (Fig 1). The Tra clade includes all of the calpains included in the analysis that contain the C2 domain characteristic of Tra calpains, including the two *Aplysia* C2 domain-containing calpains, AplTra1 and AplTra2 (Fig 3A). The original Tra calpain likely diverged at the base of the metazoan lineage as Tra family members were identified in ancient metazoan phyla such as placazoa (*Trichoplax*), but not in the sister groups to metazoans, the filastereans (*Capsaspora*) and choanoflagellates (*Salpingoeca*), or in the fungi (*Histoplasma*) (Fig 3A). Interestingly, the ctenophore (*Mnemiopsis*), that is likely at the very base of the metazoan lineage [52] (S1 Fig), has a truncated calpain whose catalytic domain falls into the Tra family, suggesting either the loss of the C2 domain in this lineage, or divergence of this family of calpains before inclusion of the C2 domain in later lineages (Fig 3A).

The phylogeny also revealed two families composed of calpains containing duplicate C2L domains (Fig 1 expanded in Fig 3B). One of these (Atypical L (Lophotrochozoan), bootstrap value: 100) includes the *Aplysia* Atypical calpain (AplAty) as well as members from *Crassostria* and *Capitella*, all of which are lophotrochozoa (Fig 3B). The second Atypical clade (Atypical V (Vertebrate), bootstrap value: 100%) is vertebrate-specific, as it contains only the CAPN-10 homologs from *Homo*, *Xenopus* and *Danio* (Fig 3B). These distinct clades, and the absence of the duplicate C2L domain structure (except in the PalB family) outside of lophotrochozoa and vertebrates, i.e. in the deuterostomes *Branchiostoma* and *Strongylocentrotus*, or the ecdysozoans *Drosophila* or *Daphnia*, suggest that the same Atypical calpain structure arose independently in lophotrochozoan and vertebrate lineages.

Interestingly, both Atypical families are grouped into a single, low-bootstrap-value (bootstrap value 36) clade with truncated calpains from lophotrochozoa (*Lottia*, *Crassostrea*, *Aplysia*), ecdysozoa (*Limulus*) and deuterostomes (*Strongylocentrotus*, *Branchiostoma*) suggesting vertebrate and lophotrochozoan Atypical calpain families may be related to a Truncated calpain family that was present in the bilaterian ancestor (Fig 3B). *Aplysia* AplTRUNC1 is a member of this bilaterian Truncated calpain family (Fig 3B). This relationship is not well-resolved in the tree, but since the phylogeny is based solely on the catalytic domain, the similarity of domain structures within the Truncated and Atypical clades support the veracity of these clades. While the bilaterian Truncated clade was also observed in the Phylip neighbor-joining analysis, this clade did not include the atypical calpains.

Interestingly, one clade emerged that was composed strictly of lophotrochozoan truncated calpains, including *Aplysia* AplTRUNC2, suggesting that these calpains make up a conserved family within the lophotrochozoa (Truncated L) (Fig 1 expanded in Fig 3C). This clade segregated weakly with several lophotrochozoan classical calpains (see below), and may be recently truncated relatives of these calpains.

It is noteworthy that vertebrate CAPN-17s also segregated with this group (Fig 3C), forming a moderately strong clade (bootstrap value 76) with CapCCAL3, AplCCal2 and AplTrunc3 and several other classical and truncated lophotrochozoan calpains. This relationship between CAPN-17 and lophotrochozoan calpains was not recapitulated in the Phylip neighbor-joining analysis, not is it seen below in the analysis of only PEF-containing calpains. (described below). However, nearly all of the lophotrochozoan CAPN-17 homologs identified in the catalytic domain-based tree were excluded from the PEF-only tree due to missing or incomplete PEF domains. Thus it is possible the fish-specific CAPN-17 family may share a common ancestor with AplCCal2 and related calpains in lophotrochozoa.

Surprisingly, calpains with PEF domains, the defining feature of classical calpains did not form a well-defined clade in this analysis. A clade of very low bootstrap value (bootstrap value 39) comprised all of the PEF-containing calpains included in the analysis, and also included the Tra family (Fig 1). Some of the truncated calpains also fell within this clade, consistent with their structure reflecting either recent truncation of a classical isoform or incomplete sequence data. AplTrunc3, for example, showed close association with AplCCal2 in the tree, consistent with recent duplication and either truncation or incomplete transcript assembly (Fig 3C). Subsets of PEF calpains segregated into smaller clades, suggesting possible subfamilies, but bootstrap values were generally too low to draw conclusions about the relationships among classical calpains. Phylogenetic and domain structure information suggest that the first PEF-containing classical calpain arose early in the pre-bilaterian metazoan lineage, as PEF-containing calpains were found in basal metazoan phyla (*Trichoplax and Amphimedon*) but not in the filastereans (*Capsaspora*) or choanoflagellates, (*Salpingoeca*), sister groups to metazoa, or in fungi (*Histoplasma*) (S1 Fig). This places the origin of the classical calpain family close in time to the emergence of the Tra family and after emergence of the PalB and SOL families. Thus the low bootstrap values associated with the classical calpain family relative to the other canonical calpain families cannot be attributed to an earlier origin, and instead is likely due to a particularly rapid rate of evolutionary change in this family.

Given the low bootstrap values associated with classical calpain-containing clades, we sought to clarify the relationships among the classical calpains by repeating the analysis using a larger region of the protease domain, and including only PEF-containing calpains.

**Fig 4:**
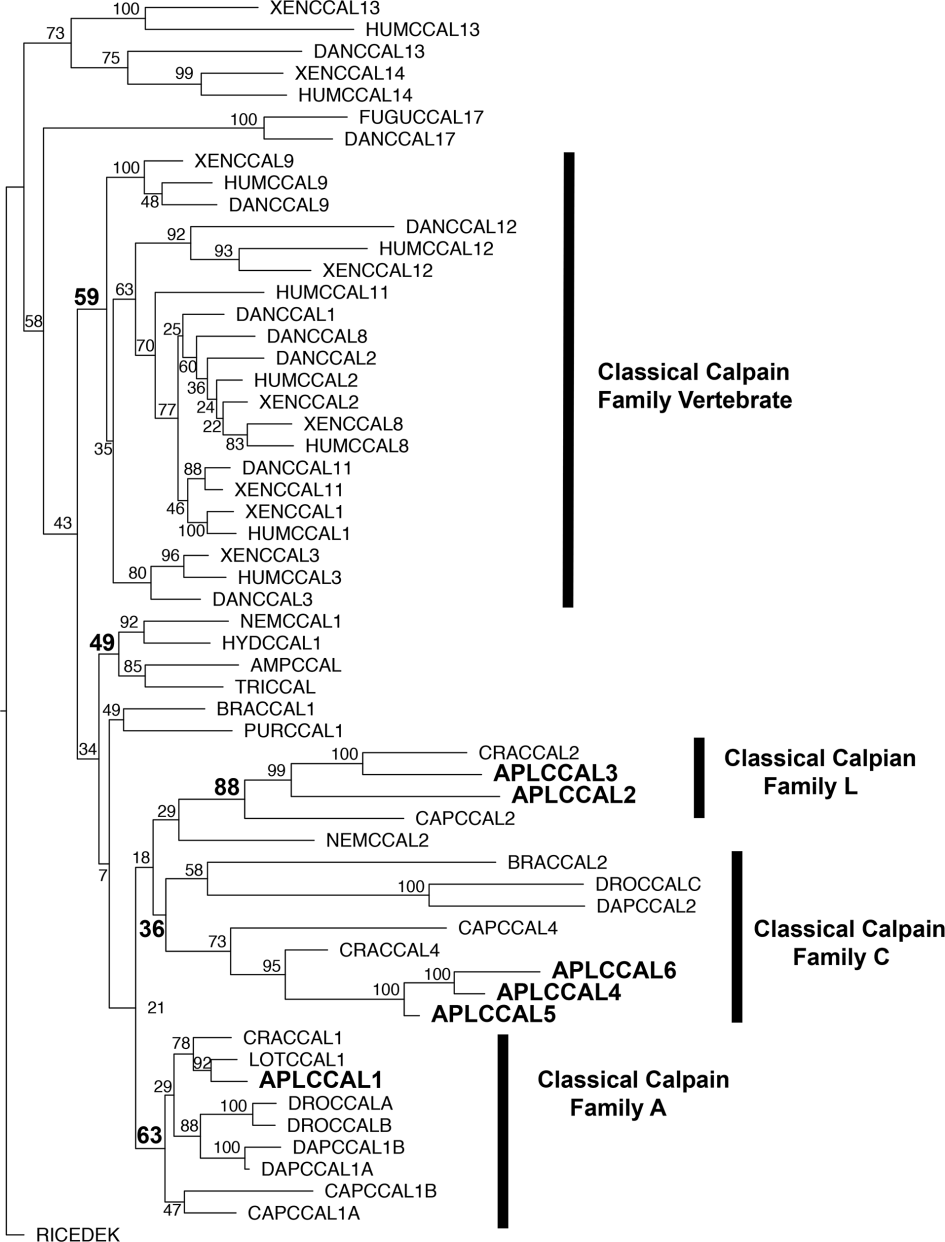
Phylogeny of classical calpain families. Species abbreviations and their phylogenetic classification and common name are detailed in Table 1. All reference numbers for sequences are in S1 Table. The analysis is described in the methods (the plot is from the RAxML analysis). *Aplysia* calpains are in larger font and bolded as are bootstrap numbers referred to in the text that define families. Families are defined by the rectangles and the family name on the right.

*Aplysia* classical calpains fell into three distinct families in this analysis (Fig 4). AplCCal1 is part of Classical calpain family A (defined by *Drosophila* Calpain A; bootstrap value 63), which includes members from both ecdysozoa and lophotrochozoa, notably including the previously characterized *Drosophila* calpains A and B. AplCCal2 and 3 are part of a lophotrochozoan-specific family, Classical calpain family L (bootstrap value 88). AplCCal 4, 5 and 6 form a strong clade (bootstrap value 100), suggesting these three calpains reflect an expansion that took place very recently in *Aplysia*’s evolutionary history. This clade in turn is part of a larger Classical calpain family C (defined by the catalytically inactive *Drosophila* calpain C; bootstrap value 36), which includes members from lophotrochozoa (*Capitella*, *Crassostrea*), ecdysozoa (*Drosophila*, *Daphnia*), and deuterostomia (*Branchiostoma)*. Thus, Classical family C is present in all three major divisions of bilaterians and presumably arose before the bilaterian ancestor. While the bootstrap value in this analysis is low, this clade was observed in both neighbor-joining and maximum-likelihood analyses with similar levels of confidence. Interestingly, while the ecdysozoan (*Drosophila*, *Daphnia*) and deuterostome (*Branchiostoma)* members of this family have lost critical catalytic residues and are thus likely to be inactive, the lophotrochozoan (*Crassostrea*, *Aplysia*, *Capitella*) members all conserve the catalytic triad seen in catalytically active calpains,

Consistent with previous reports [2, 53] the placement of the vertebrate calpains in the classical calpain phylogenetic tree also suggests recent expansion from a small number of ancestral classical calpains. Although the bootstrap value was fairly low (bootstrap value 59), most of the vertebrate classical calpains (CAPN-1, 2, 3, 8, 9, 11, 12) formed a single clade, which did not include any non-vertebrate calpains (Vertebrate calpains, bootstrap value 59), in the maximum-likelihood phylogeny. An identical clade was recently reported in a phylogenetic analysis of eukaryotic and bacterial calpains [2]. A similar clade emerged in the Phylip neighbor-joining analysis, with the exception that the CAPN-12 homologs were excluded and formed a separate clade. Although there is evidence that CAPN-3 and 9 were both present in the last common vertebrate ancestor [53], our phylogeny and others [2] suggest that these, and vertebrate CAPN-1,2,8,11 and possibly 12 result from proliferation of a single ancestral calpain that was present in early chordates. Vertebrate CAPN-13 and 14 formed a distinct clade of moderate strength (bootstrap 73). This is consistent with previous analyses [2], one of which indicated CAPN-13 and 14 were produced by the duplication, in early lobe-finned fish, of a calpain that was present in the last common vertebrate ancestor [53]. CAPN-17s, which are only found in fish [53], did not form a strong clade with other vertebrate or non-vertebrate calpains, and thus the origin of this isoform is unclear although our catalytic domain-based phylogenetic analysis suggests that CAPN-17s may share a common ancestor with AplCCal2 and its lophotrochozoan homologs (Fig 3C). Our results are consistent with a recent analysis of vertebrate calpains, which also suggested that the lineages of CAPN-12,13/14 and 17 are distinct from the rest of the vertebrate calpains [53]

Our analysis did not resolve the relationships among the non-vertebrate Classical calpain families A, L and C, and the vertebrate calpains. Indeed the critical basal deuterostome members examined (*Brachiostoma*, *Strongylocentrotus*) either segregated with non-vertebrate families (BRACCAL2) or formed their own separate family (BRACCAL1 and PURCCAL1) with no obvious link to any of the vertebrate lineages. In summary, there have been major expansions of classical calpains in both vertebrates and lophotrochozoa from a small number of ancestral classical calpains, but the orthology relationships between lophotrochozoan and vertebrate classical calpain subfamilies are unclear.

### Multiple variants of AplCCAL1

**Fig 5:**
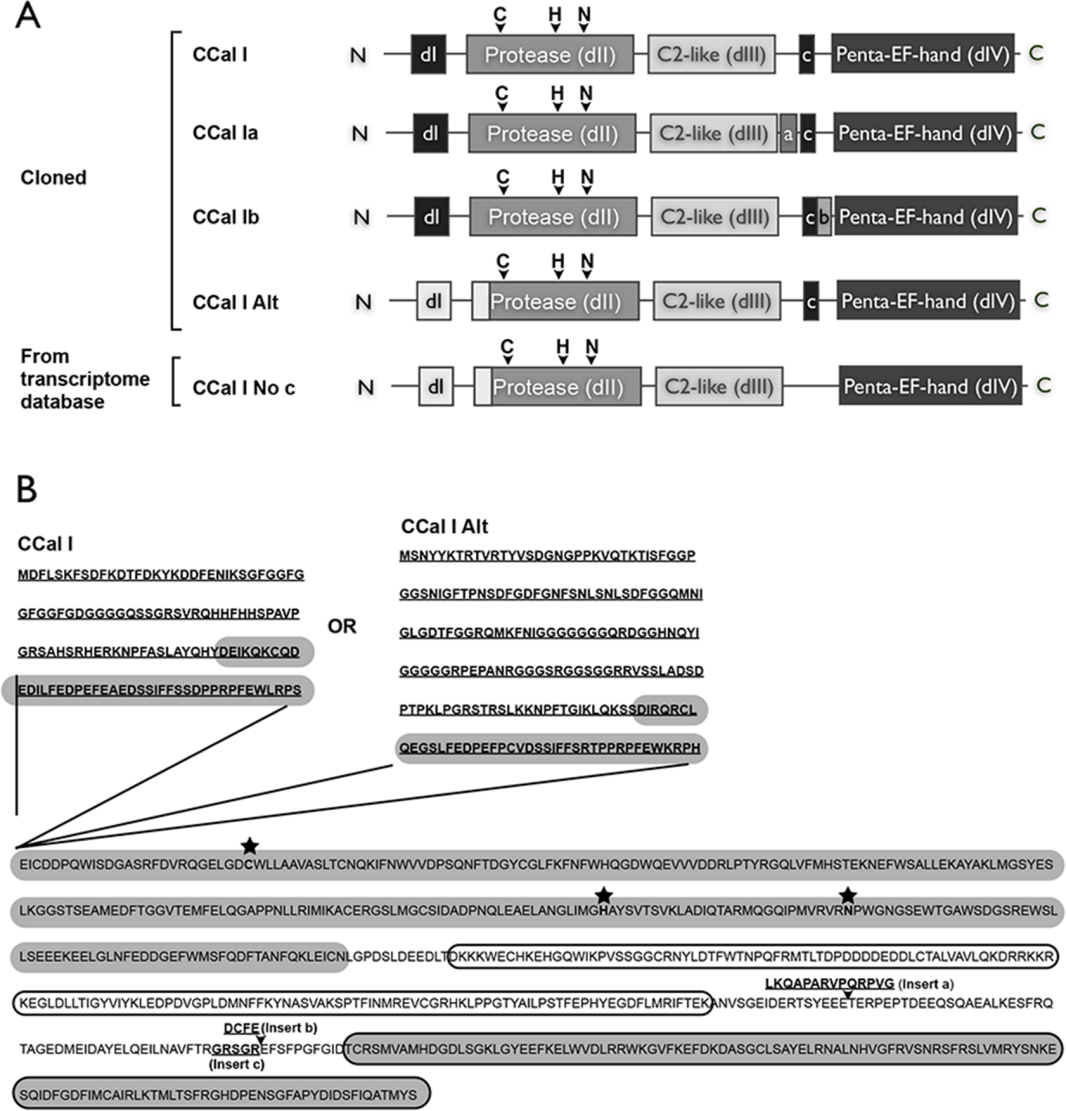
Variants of AplCCal1. (A) Schematic of the domain structure of the nervous system variants of AplCCal1. Splice inserts a, b, and c, and the alternative N-termini are indicated by shaded blocks. The known calpain domains are shown. The positions of the catalytic triad: cysteine(C), histidine (H) and asparagine (N) are shown. (B) Amino acid sequence of the AplCCal1 variants. Splice inserts a, b, and c, and the alternative N-termini are indicated. The catalytic residues are marked with stars. Domains (catalytic (shaded), C2L (outlined), PEF (outlined and shaded)) are shown in ovals. Note that the catalytic domain starts in the alternative N-terminal exons.

The evidence of proliferation of classical calpains in invertebrates like *Aplysia* raises the possibility of novel mechanisms of classical calpain regulation, yet other than in *Drosophila* [54, 55], invertebrate calpains have not been characterized biochemically. We therefore characterized the catalytic activity of AplCCal1, the *Aplysia* classical calpain implicated in PKM formation during synaptic plasticity [17, 18]. While amplifying AplCCal1 from nervous system cDNA for cloning purposes, we found a number of alternative transcripts. These included three splice variants containing either insert a (LKQAPARVPQRPVG), insert b (DCFE), or neither insert in the region between the C2L domain and the PEF domain (Fig 5A and 5B). Sequence from the *Aplysia* Transcriptome Assembly (www.Aplysiagenetools) further indicated an additional splice insert c (GRSGR), also in the region between the C2L and PEF domains, which was present in all of our clones but absent from some transcripts in this database. The transcriptome assemblies also contained transcripts with an alternative N-terminal suggesting the existence of two distinct transcriptional start sites. The presence of this alternative N-terminal transcript in nervous system cDNA was confirmed by PCR and a construct was generated with this alternative N-terminal sequence. Thus, there are four distinct alternative exons/transcriptional start sites suggesting as many as 16 possible AplCCal1 variants if these are independently used. Below, we used AplCCal1a, (containing the a and c exons), AplCCal1b (containing the b and c exons), and AplCCal1alt (containing the alternative N-terminal and c exon) (Fig 5A and 5B).

### Characterization of Calpain activity of AplCCAL1

We expressed and purified His-tagged recombinant forms of the cloned variants of AplCCal1 in Spodoptera frugiperda (SF9) insect cells using a baculovirus expression system. Since *Aplysia* are marine mollusks with a basal temperature of about 15°C, this allows expression at a temperature closer to native than bacterial or mammalian expression systems. We examined the Ca^2+^-induced activity of purified recombinant AplCCal1 against casein, a well-established substrate for different classical calpain isoforms [56] including invertebrate classical calpains [55]. For comparison we also tested the activity of heterodimeric CAPN-1 purified from porcine erythrocytes (Calbiochem). Mammalian classical calpains are known to autolyse toward the N-terminus upon activation by Ca^2+^ and then rapidly self-degrade *in vitro* [57]. Thus we monitored calpain autolysis as an independent indicator of calpain activation.

**Fig 6:**
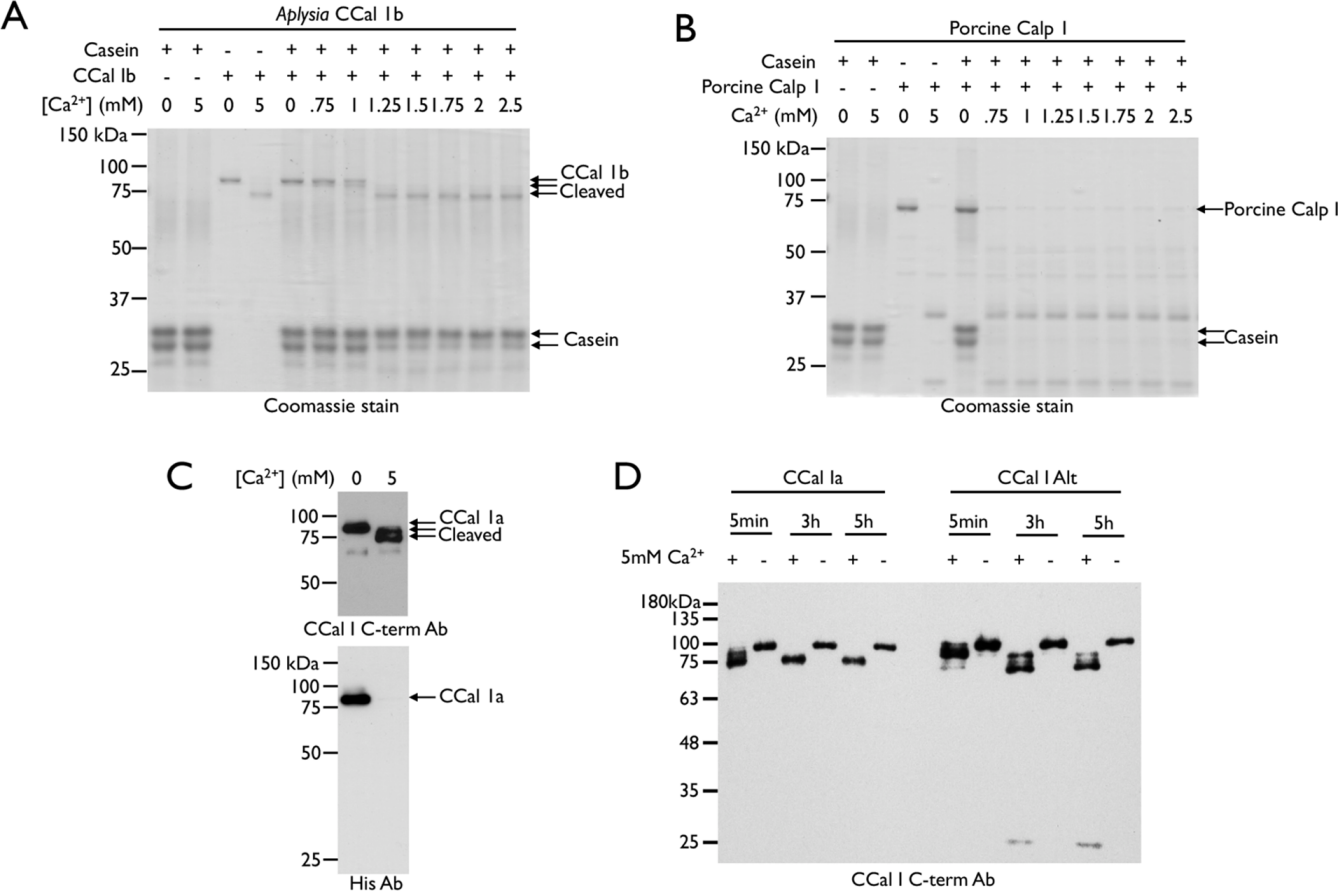
Autolysis of Aplysia AplCCal1 produces a stable cleavage product. Coomassie-stained gel representing (A) *Aplysia* AplCCal1b (80ng/ul) and (B) Porcine classical CAPN-1 (80ng/ul) incubated with casein (225ng/ul) for 30 min at various Ca^2+^ concentrations. Twenty-eight microliters of the reaction was loaded. (C) *Aplysia* AplCCal1a (290ng/ul) was incubated with or without Ca^2+^ for 30 min. Thirty-one microliters of the reaction was subjected to immunoblot with an antibody against the N-terminal His-tag, stripped and reprobed with an AplCCal1 C-terminal antibody. (D) AplCCal1a or AplCCal1alt (50ng/ul) was incubated in the presence or absence of Ca^2+^. At the indicated timepoints, 25ul of each reaction was removed and inactivated by addition of 5X Laemmli sample buffer. Samples were subjected to immunoblot with an antibody against the AplCCal1 C-terminus.

AplCCal1 did not undergo complete Ca2+-dependent autolytic degradation, but instead underwent a mobility shift upon incubation with 0.5–1.25mM Ca2+, consistent with limited autolysis (4 independent experiments with AplCCal1b and 1 experiment with AplCCal1alt; Fig 6A). In contrast, porcine CAPN-1 completely degraded itself under similar conditions (Fig 6B). For AplCCal1, a diminution of the casein bands became evident at the same Ca^2+^ concentration at which N-terminal cleavage occurred, although casein cleavage was incomplete. In contrast, porcine CAPN-1 completely eliminated the casein bands (2 independent experiments with AplCCal1a, 2 independent experiments for AplCCal1b and 1 experiment with AplCCal1alt, Fig 6A and 6B). No autolytic cleavage was seen with purified AplCCal1 when the catalytic cysteine was converted to a serine (S3 Fig) demonstrating that cleavage was not due to a co-purified protease.

Further experiments demonstrated the Ca^2+^-dependent mobility shift of AplCCal1 was due to N-terminal cleavage as immunoreactivity was lost with the antibody to the His-Tag located at the N-terminal, but not with an antibody raised to the C-terminal of AplCCal1 (2 independent experiments with AplCCal1b, 1 experiment with AplCCal1a, Fig 6C). This demonstrates that *Aplysia* CCal1 undergoes N-terminal proteolysis upon activation, similar to previously characterized mammalian and *Drosophila* classical calpains (55, 63).

The fact that Ca^2+^ triggered complete N-terminal cleavage of *Aplysia* AplCCal1, but no detectable self-degradation and relatively weak casein cleavage, suggests that N-terminal autolysis may inactivate the calpain. To further examine this, we performed a time-course experiment to observe if the cleaved AplCCal1 was stable over time. Since the autolysis sites are presumably different between the two alternative N-terminal variants, both were examined. Over the course of a five-hour reaction, both N-terminal variants underwent one or more intermediate cleavage events that were not stable, before ultimately being converted into a stable N-terminally truncated form (3 independent experiments for AplCCal1alt and 3 for AplCCal1a; Fig 6D). We observed a difference in kinetics between the alternative N-terminal variants, with AplCCal1alt being cleaved considerably slower than AplCCal1a, and there was also a small difference in the size of the final, stable cleaved form. This is consistent with N-terminal autolysis ultimately inactivating both *Aplysia* AplCCal1 N-terminal variants despite differences in the sites of autolysis and the rate at which autolysis occurs.

**Fig 7:**
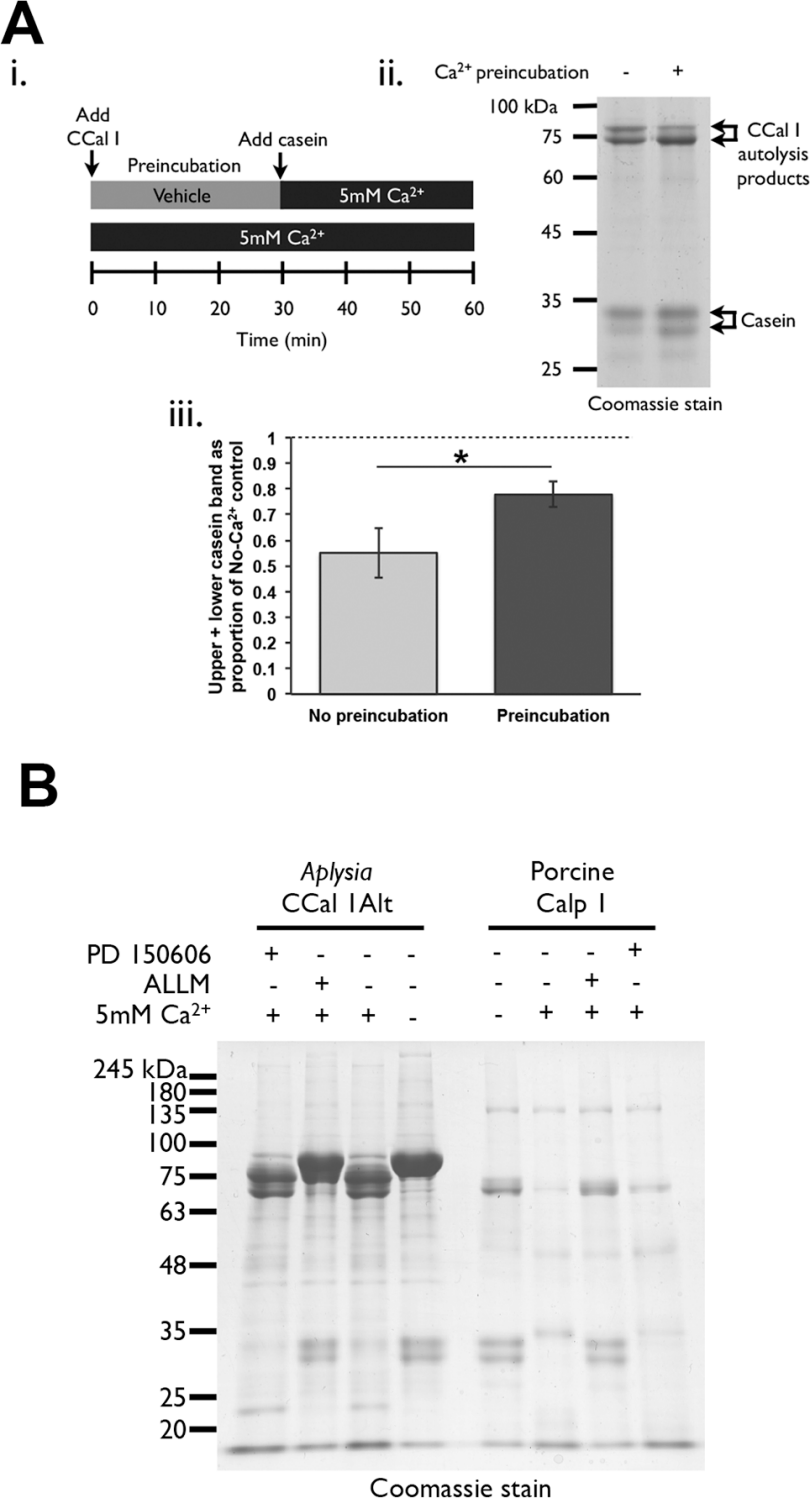
Characterization of the sensitivity of AplCCal1 activity to N-terminal cleavage and calpain inhibitors. (A) AplCCal1 (200ng/ul) was pre-incubated for 30min with or without Ca^2+^ before addition of casein substrate (205ng/ul) with or without Ca^2+^, then incubated 30min before 33ul of the reaction was subjected to gel electrophoresis and Coomassie staining. (i) Schematic of experimental design. (ii) Representative Coomassie stained gel. (iii) Quantification of combined upper and lower casein bands, expressed as a proportion of a control that was never exposed to Ca^2+^. A one-tailed t-test for paired samples yielded p<0.05, represented by an asterisk (*). Data from 3 independent experiments. Error bars show SEM. (B) After a 20min preincubation with 100uM PD-150606, ALLM or vehicle, AplCCal1alt (325ng/ul) or porcine CAPN-1 (116ng/ul) and casein (195ng/ul) were incubated in the presence or absence of 5mM Ca2+, and in the presence or absence of 100uM PD-150606, ALLM or vehicle for one hour at room temperature. Thirty-two microliters of each reaction was subjected to SDS-PAGE and Coomassie staining. AplCCal1alt was used at higher concentrations than porcine calp-1 to compensate for its lower activity against casein.

If Ca^2+^-induced N-terminal cleavage inactivates AplCCal1 then AplCCal1 that has already autolysed should be unable to cleave casein. To test this prediction, AplCCal1 was pre-incubated for 30min with 5mM Ca^2+^ to induce autolysis, or without Ca^2+^ as a control, and then combined with casein in a final concentration of 5mM or 0mMCa^2+^ and incubated for 30min. Preincubation with Ca^2+^ resulted in greater autolysis, but reduced casein cleavage (Fig 7A). This is consistent with N-terminal autolysis inactivating *Aplysia* AplCCal1.

We next tested whether pharmacological calpain inhibitors could block *Aplysia* AplCCal1 N-terminal cleavage and casein degradation *in vitro*. We used ALLM (Calpain inhibitor II), a competitive inhibitor [58] which has been found to block site-specific sensitization in *Aplysia* [14] and to mimic the inhibitory effect of dominant negative AplCCal1 on plasticity-related PKC cleavage in neurons [17]. We also used the noncompetitive inhibitor PD150606, which targets the PEF domain [59], and which has been used to infer that calpains play no role in long-term facilitation in *Aplysia* [60]. It is important to note that although these inhibitors have been used to examine the role of calpains in *Aplysia*, their effectiveness against *Aplysia* calpains has never been tested before. In three independent experiments, ALLM was effective at blocking autolysis and casein degradation for both porcine CAPN-1 and AplCCal1alt, supporting the calpain-dependence of these events (Fig 7B). In contrast, PD150606 was a poor inhibitor of both porcine and *Aplysia* calpains in the same experiments, only slightly reducing porcine CAPN-1 autolysis and having no effect on its degradation of casein, and having no detectable effect on AplCCal1alt. The lack of effect of PD150606 against *Aplysia* AplCCal1 may be due in part to insusceptibility of the *Aplysia* calpain to this inhibitor, although our results indicate PD150606 is also a much weaker inhibitor of porcine CAPN-1 than ALLM is, and the effectiveness of PD150606 against vertebrate calpains has recently been challenged [61].

**Fig 8:**
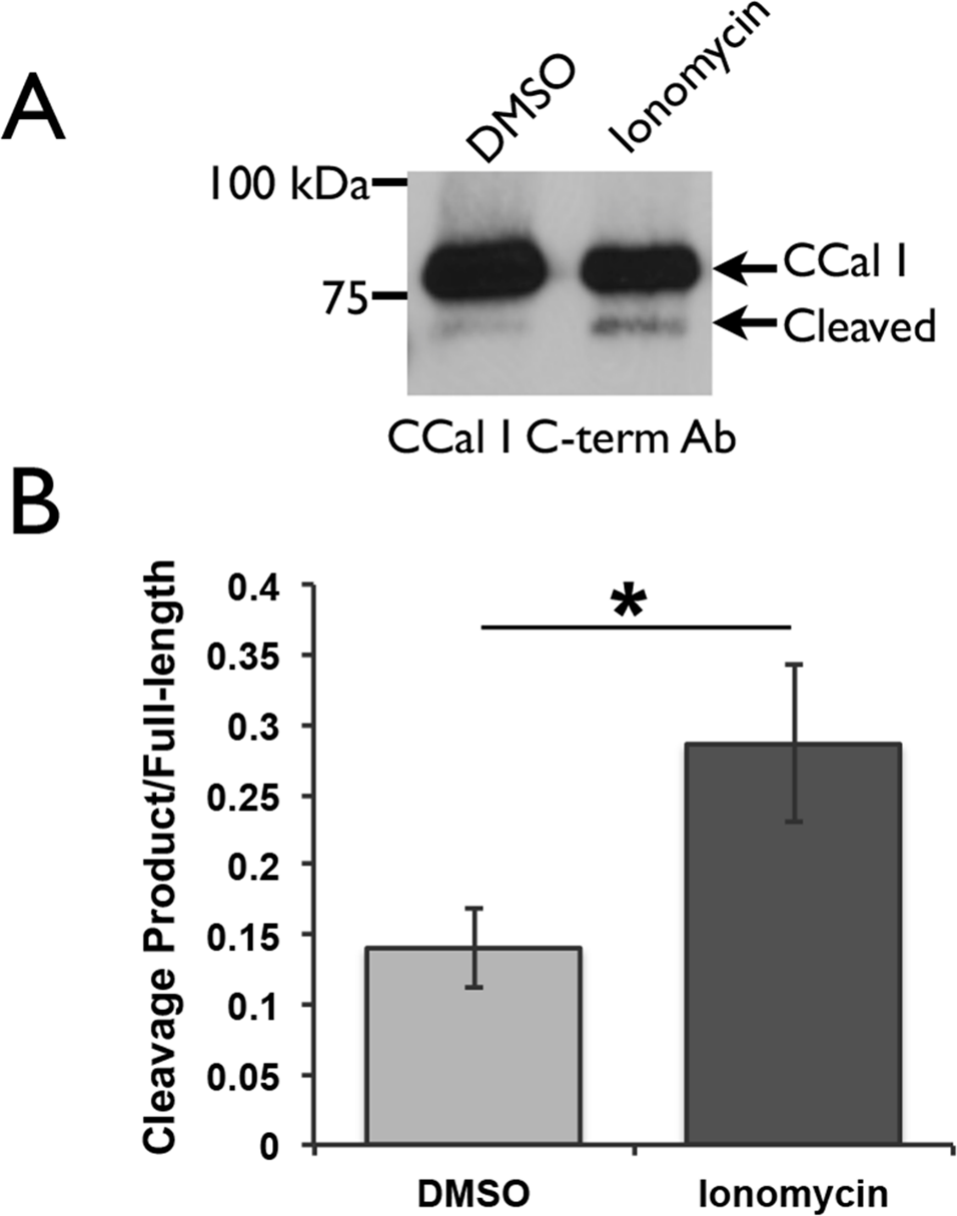
AplCCal1 autolyses *in vivo*. (A) Desheathed paired pleural-pedal ganglia were treated for 20min with 100uM ionomycin or vehicle and then homogenized and subjected to western blot with an antibody against the C-terminal of AplCCal1. (B) Quantification of data from 4 independent experiments as shown in (A). A one-tailed t-test for paired samples yielded p<0.05, represented by an asterisk (*). Error bars show SEM.

To explore whether the N-terminal autolysis of AplCCal1 observed *in vitro* might also occur in neurons, paired pleural-pedal ganglia were extracted from *Aplysia*, treated with either vehicle or ionomycin to increase intracellular calcium, then homogenized and subjected to western blot with an antibody against the AplCCal1 C-terminal. Ionomycin consistently increase the intensity of a band slightly below full-length calpain, consistent with N-terminal autolysis in neurons at high calcium levels (Fig 8).

### AplCCal11 can cleave PKCs into PKMs

**Fig 9:**
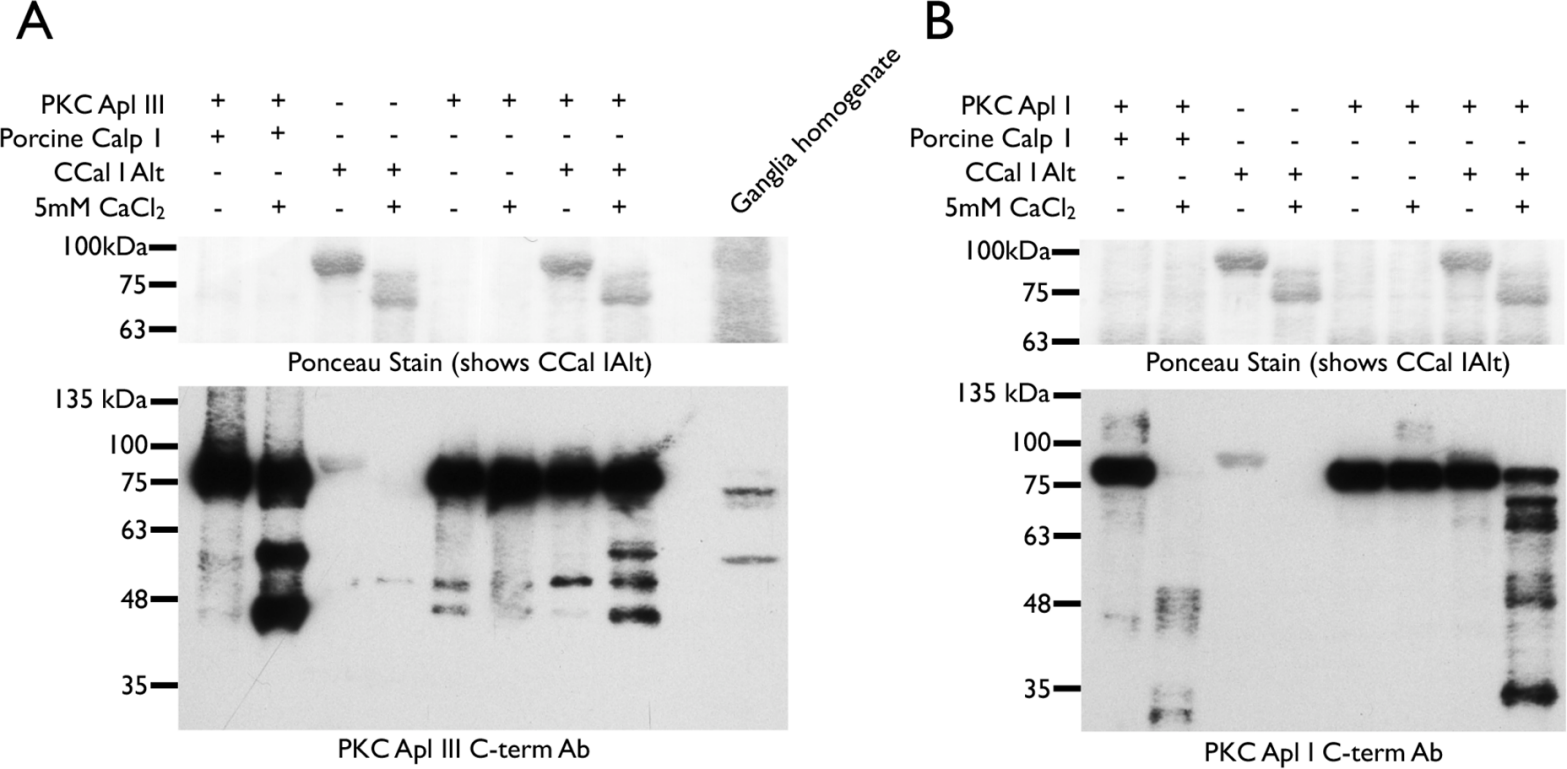
*Aplysia* AplCCal1 cleaves PKC Apl I and PKC Apl III. (A) Porcine calp-1 (85ng/ul) or *Aplysia* AplCCal1alt (855ng/ul) were incubated with PKC Apl III (80ng/ul) at room temperature for 1h (porcine calp-1) or 3h (*Aplysia* CCal1alt) in the presence or absence of Ca^2+^. AplCCal1alt alone and PKC Apl III alone controls were also included. After incubation, 17ul samples were run alongside *Aplysia* ganglia homogenate on SDS-PAGE gel, transferred to nitrocellulose membrane, ponceau stained and probed with a PKC Apl III C-terminal antibody. Similar cleavage was observed in three independent experiments for AplCCal1b, one for AplCCal1a and two for AplCCal1alt (B) Porcine calp-1 (85ng/ul) or *Aplysia* AplCCal1alt (855ng/ul) were incubated with PKC Apl I (550ng/ul) at room temperature for 1h (porcine calp-1) or 3h (*Aplysia* AplCCal1alt) in the presence or absence of Ca^2+^. AplCCal1alt alone and PKC Apl I alone controls were also included. After incubation, 17ul samples were subjected to SDS-PAGE, transferred to nitrocellulose membrane, ponceau stained and probed with an antibody directed against the PKC Apl I C-terminus. Similar cleavage was observed in three independent experiments with AplCCal1alt and one with AplCCal1b.

*Aplysia* AplCCal1 has been implicated in cleavage of PKC Apl I and PKC Apl III in different synaptic plasticity paradigms [17, 18] and mammalian CAPN-1 has been shown to cleave these PKCs to form PKMs *in vitro* [14, 35], but direct cleavage of PKCs by AplCCal1 has not yet been demonstrated. To confirm that AplCCal1 is capable of cleaving PKCs into PKMs we incubated AplCCal1 with purified recombinant PKC Apl I or PKC Apl III in the presence or absence of calcium (Fig 9). For these experiments we used AplCCal1alt since it inactivates more slowly and thus would be expected to show a higher activity against exogenous substrates. The calpain was used at high concentrations to overcome its inefficiency against substrates other than itself. AplCCal1alt cleaved both PKC Apl I and PKC Apl III in a calcium-dependent manner and the cleavage products were similar to those observed with porcine CAPN-1, although not identical, and several additional cleavage products were present for PKC Apl I. For PKC Apl III the two major cleavage products produced by mammalian CAPN-1 have been previously characterized. The upper band represents cleavage at the beginning of the hinge domain between the regulatory and catalytic domain of the PKC, while the lower band represents cleavage in a nervous-system-enriched splice cassette just C-terminal to the hinge domain and at the beginning of the catalytic domain [35]. Thus both bands are thought to represent PKMs.

Ganglia homogenate run alongside cleaved PKC Apl III revealed a putative endogenous PKM similar but not identical in mobility to the higher molecular weight PKM produced by porcine CAPN-1 and AplCCal1alt (Fig 9A). No PKM Apl I was detected in ganglia homogenates (data not shown).

**Fig 10:**
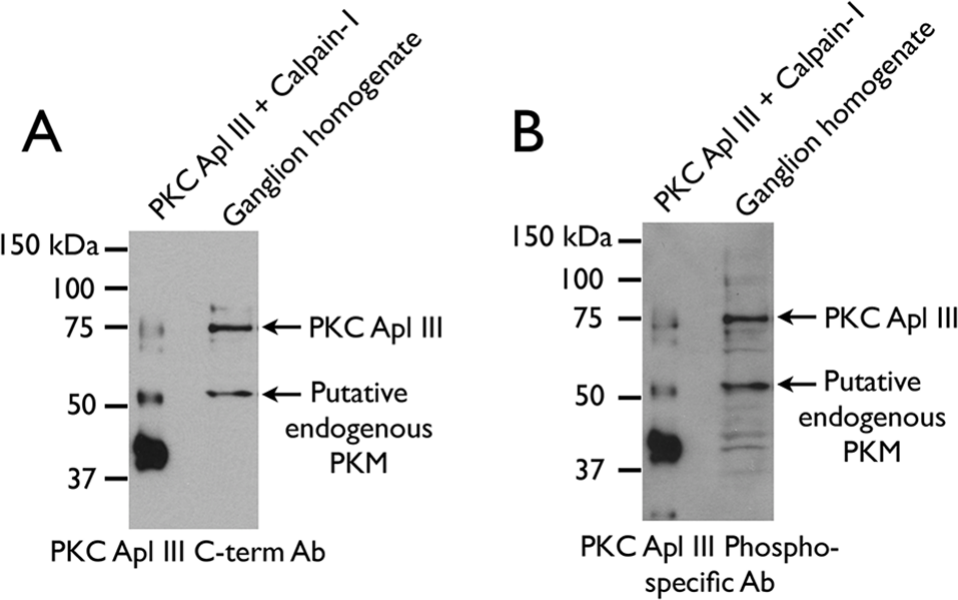
Characterization of endogenous PKM in homogenates. Antibodies against the PKC Apl III C-terminus (A) and B) PDK phosphorylation site (B) were used to probe membranes containing ganglion homogenate. For comparison, the neighboring lane contains purified PKC Apl III that has been incubated with mammalian CAPN-1 to induce cleavage.

To confirm the identity of the putative PKM Apl III band detected with the PKC Apl III C-terminal antibody in ganglia homogenates, we used an independent, previously characterized phosphospecific antibody against the phosphoinositide-dependent kinase 1 (PDK) site near the C-terminus of PKC Apl III, which would also be expected to detect PKM Apl III [35]. In these blots, ganglia homogenates were run alongside PKM produced *in vitro* by cleavage with porcine CAPN-1 for band size comparison. The phosphospecific antibody recognized a band of similar size to the putative PKM detected with the PKC Apl III C-terminal antibody in both ganglia homogenate and in the product of the *in vitro* calpain-cleavage assay (Fig 10A and 10B). This increases the confidence that the putative PKM in ganglia homogenate is a bona fide cleaved product of PKC Apl III. Interestingly, while the predominant PKM produced by *in vitro* cleavage is the lower molecular weight form, reflecting cleavage in a nervous system-enriched splice insert, the predominant PKM in homogenates is closer in size to the higher molecular-weight form produced by cleavage at the beginning of the hinge domain of PKC Apl III. Whether this is due to instability of the low-molecular-weight PKM *in vivo*, or to factors restricting its formation, is not clear at this time.

## Discussion

### Evolutionary analysis of calpain families

We have identified a total of fourteen calpain encoding genes in *Aplysia*, including 1 SOL, 1 PalB, 2 Tra, 6 classical calpains, 1 atypical calpain and 3 truncated forms (Table 2). Consistent with previous observations [2, 53] our phylogenetic analysis of calpains indicates that SOL and PalB calpains are ancient, highly conserved families that arose before metazoa, while Tra and Classical calpains arose later, near the base of the metazoan lineage. Classical, PEF domain-containing, family members are the most numerous and their phylogenetic relationships are more difficult to parse than the other canonical calpain families suggesting a unique propensity for proliferation and rapid change.

**Table 2:** *Aplysia* calpains.

Aplysia Calpains	GI	Calpain family	Comments
APLCCAL1	XP_012942076.1	Classical family A	N-terminal auto-inactivation. Required for intermediate [17] and associative LTF [18]
APLCCAL2	XP_005093816.2	Classical family L	
APLCCAL3	XP_012940412.1	Classical family L	
APLCCAL4	XP_012942068.1	Classical family C	
APLCCAL5	XP_012942074.1	Classical family C	
APLCCAL6	XP_005105754.2	Classical family C	
APLSOL	XP_012936257.1	SOL family	Required for non-associative LTF [18]
APLPALB	c125304_g3_i1 len = 3129	PalB family	
APLTRA1	c103226_c0_seq3 length = 4878	Tra family	
APLTRA2	c120955_g1_i1 length = 2580	Tra family	
APLATY	XP_005089461.1	Atypical L family	
APLTRUNC1	XP_005105403.1	Truncated family	
APLTRUNC2	c6_g1_i3 len = 3371	Truncated L family	
APLTRUNC3	XP_012935411.1	Classical family L AplCCAL2	Recently diverged from AplCCAL2

The name, accession number, family based on our phylogenetic analysis, and any other information are shown for each of the 14 calpain family members identified from *Aplysia*.

Our phylogeny revealed several novel calpain families. These included two independent clades composed solely of Atypical calpains containing tandem C2L domains in vertebrates (CAPN-10 homologs) and lophotrochozoa (AplAty homologs). Both may be related to a Truncated calpain family that is conserved across a range of bilaterian phyla, although bootstrap values were weak for these relationships. A previous catalytic domain-based phylogenetic analysis concluded that vertebrate CAPN-10 belongs to a clade that diverged in early metazoa, but did not report whether all members of this clade possessed duplicate C2L domains [2]. It would be interesting to explore the possibility that early bilaterian ancestors of CAPN-10 and the lophotrochozoan Atypical calpains may have been truncated calpains with only one C2L domain.

The analysis also revealed a distinct lophotrochozoa-specific Truncated calpain family (Truncated L), which includes AplTrunc2, and which may have arisen through truncation of an ancestral lophotrochozoan classical calpain. Calpains with a similar structure to the truncated forms described here have also been identified in several other eukaryotic groups besides metazoa, and this structure is thought to have evolved multiple times in eukaryotes [1]. It is unclear whether any of these are orthologous to any of the truncated calpains we identified in metazoa. The activity, regulation and function of truncated calpains is an open question.

Phylogenetic analyses limited to classical calpains indicate that the large number of classical calpains in *Aplysia* (6 isoforms) and in vertebrates (9 isoforms in humans) result mainly from recent expansions, and not from descent from numerous shared ancestral bilaterian calpains. The actual number of shared ancestral bilaterian classical calpains, however, remains unclear. The *Aplysia* classical calpains fell into three separate families (L, A and C) in the classical calpain phylogeny. While family L was lophotrochozoa-specific, family A contained ecdysozoan members and family C included both deuterostome and ecdysozoan members, suggesting there may have been two classical calpains in the bilaterian ancestor. The vertebrate classical calpains similarly fell into three or four vertebrate-specific families (CAPN-13/14, CAPN-1/2/3/8/9/11/possibly 12, and CAPN-17) corroborating previous descriptions of classical calpain expansions in the vertebrate lineage [2, 53]. However, the relationship of these vertebrate families to one another and to invertebrate classical calpains could not be clearly determined through sequence similarity. Thus it is possible that rapid evolutionary change has obscured the phylogenetic relationships between classical calpain subfamilies shared by vertebrates and invertebrates. Indeed, the tendency of vertebrate classical calpains to proliferate and diverge functionally has complicated comparative studies of calpains between vertebrate species [53].

The classical calpain analyses (Fig 2) also provide potentially valuable insight into the relationships between *Aplysia* calpains and the previously characterized *Drosophila* calpains. The plasticity-related *Aplysia* calpain AplCCAL1 is homologous to CalpA and CalpB, the only catalytically active classical calpains in Drosophila.

### AplCCal1 is inactivated by N-terminal cleavage

Given the obscurity of orthology relationships of the *Aplysia* calpains to the better studied vertebrate counterparts, it is important to characterize the activation of calpains in order to understand how they play a role in synaptic plasticity in *Aplysia*. We discovered four alternative transcripts of the *Aplysia* plasticity-related classical calpain, *Aplysia* AplCCal1 expressed in the nervous system and investigated the catalytic activity of several of these variants.

AplCCal1 showed weak activity against casein and PKC *in vitro*, despite complete Ca^2+^-dependent N-terminal autolysis of the calpain. Autolysis was triggered by Ca^2+^ concentrations in the 0.5–1.25mM range, comparable to the previously reported Ca^2+^ requirement of mammalian CAPN-2 and higher than those of mammalian CAPN-1 [62]. The blunted activity toward substrates appears to be due to rapid autoinactivation by N-terminal autolysis of the calpain, as the N-terminally cleaved AplCCal1 does not go on to degrade itself as porcine CAPN-1 does, and induction of autolysis by pre-incubation with Ca^2+^ results in reduced cleavage of subsequently added casein. Autolysis was blocked by the calpain inhibitor ALLM. Interestingly, the site and rate of autolysis are different for the alternative N-terminal variants, but a stable, inactive product is produced from both variants. How autolysis inactivates the enzyme is not clear, although the fragment removed is large enough to potentially include part of the catalytic domain. It will be important in future to determine the exact site of cleavage.

The autoinactivation through N-terminal autolysis sets *Aplysia* classical calpain apart from the better-studied mammalian classical calpains CAPN-1 and 2 and Drosophila CalpA and B. The mammalian calpains have been demonstrated to undergo Ca^2+^-induced N-terminal autolysis *in vitro* that does not inactivate them, but rather reduces the Ca^2+^-requirement for activity [57, 63, 64], and this is rapidly followed by complete self-degradation [57]. *Drosophila* CalpA similarly self-degrades, while CalpB undergoes N-terminal autolysis that produces a fragment that is stable for at least 10min, and which is fully active [65]. Ionomycin-induced N-terminal autolysis of Drosophila CalpB has been observed *in vivo* [66] but whether N-terminal autolysis and/or self-degradation play a role in calpain regulation *in vivo* is not clear [67].

While we did observe autolysis in *Aplysia* nervous system tissue after treatment with the Ca^2+^ ionophore ionomycin, this appeared to be a minor extent of cleavage compared to the complete autolysis observed *in vitro*. It is possible that an unknown binding partner serves to stabilize AplCCal1 and limit autolysis *in vivo*. PEF domains usually form either homo- or hetero-dimers through the fifth EF-hand [68]. While *Drosophila* CalpB is thought to act as a monomer [55], mammalian CAPN-1 and 2 each form heterodimers with a small subunit [69], CAPN-3 and 13 form homodimers [70], and CAPN- 8 and -9 form a heterodimer with one another [71, 72]. Thus, even in the recently expanded chordate clade of classical calpains a large range of hetero- and homodimerization occurs. It should be noted that the small subunit has been suggested to be derived from duplication and divergence of the vertebrate CAPN-3 gene [73] and there is no evidence for a small subunit in invertebrates. It will be interesting in the future to determine if *Aplysia* AplCCal1 forms homodimers or heterodimers with the other *Aplysia* classical calpains, and whether this limits autolytic inactivation *in vivo*.

Finally, while we confirmed that ALLM, an inhibitor used to test the role of calpain in memory and plasticity in *Aplysia*, is an effective inhibitor of *Aplysia* AplCCal1, we were unable to detect any inhibitory effect of PD150606 on AplCCal1. This complicates interpretation of a previous study where the lack of effect of PD150606 was taken as evidence that calpains were not required for LTF in *Aplysia*[60].

### AplCCAL1 can cleave PKCs into PKMs

Although *Aplysia* AplCCal1 has been implicated in synaptic plasticity and PKC cleavage based on the effects of a dominant negative construct, our results establish for the first time that AplCCal1 can cleave PKC Apl I and III *in vitro*. This cleavage is inefficient relative to that catalyzed by porcine CAPN-1, but produces cleavage products of similar sizes to those of the porcine CAPN-1.

We identified a putative PKM Apl III band in ganglion homogenates, which was recognized by two antibodies against distinct epitopes in the PKC Apl III catalytic region. This putative PKM was similar in size but not identical to the higher molecular weight PKM bands produced by porcine CAPN-1 and *Aplysia* AplCCal1 cleavage of PKC Apl III *in vitro*. Interestingly, the major PKM band produced by calpain cleavage *in vitro* is actually a lower molecular weight form that is either absent or underrepresented in ganglia homogenate. This observation suggests either instability of the smaller PKM in ganglia, or that cleavage is restricted in cells in ways that it is not restricted in our *in vitro* assays.

### Implications for synaptic plasticity and memory formation

Calpains are required for the induction of synaptic plasticity and memory in both. rodents and *Aplysia* [9, 13, 18, 37, 43, 74]. Thus, it was somewhat surprising that the vertebrate CAPN-1 and -2 that are implicated in memory formation are products of a recent expansion and that AplCCAl1 is not a clear orthologue of these calpains. Many of the products of the vertebrate expansion seem to have taken on tissue-specific roles (i.e. CAPN-3 in muscle[75] and CAPN-8/9 in gastrointestinal tissues [71]) and it may be that CAPN-1 and -2 have retained more of an ancestral role in synaptic plasticity. In contrast, the SOL family shows strong conservation. In *Aplysia*, the SOL calpain is implicated in the formation of non-associative forms of synaptic plasticity [18, 43] and it will be interesting to see if this is a conserved role of this calpain.

While a dominant negative form of AplCCaL1 blocked several distinct aspects of synaptic plasticity in *Aplysia* [17, 18, 43], it does not rule out roles for the other *Aplysia* classical calpains. If AplCCaL1 heterodimerizes with one of the other *Aplysia* classical calpains then a calpain-inactive form of AplCCaL1 could act as a dominant negative against the partner calpain. Moreover, while AplCCaL1 blocked the formation of long-term synaptic plasticity, neither the dominant negative AplCCaL1 nor dominant negative AplSOL blocked the maintenance of long-term facilitation, despite the continued requirement for PKMs [18]. It is possible that one of the other *Aplysia* calpains is important in the maintenance phase of memory formation.

### Conclusions

In conclusion, we have identified 14 calpains in the *Aplysia* transcriptome/genome and while the orthology relationships of the TRA, SOL and PalB families are relatively strong and straightforward, the classical calpain families are difficult to parse due to rapid proliferation and divergence in this family. It is clear, however, that the large number of classical calpains in mollusks and vertebrates result primarily from recent, independent expansions from one, or a small number of, shared ancestral bilaterian calpain(s). Our phylogenetic analyses also revealed new atypical and truncated families in lophotrochozoa, and suggested a novel truncated family that is conserved in both deuterostomes and protostomes. We demonstrated that *Aplysia* AplCCal1, the classical calpain implicated in synaptic plasticity, could cleave PKCs into PKMs, but *in vitro* this activity was limited by inactivation through N-terminal autolysis.

## Supporting information

S1 Table Accession numbers. Accession numbers for all sequences used in the phylogenetic analysis. For sequences downloaded from transcriptome analysis the most recent download information is given. Sequences are arranged by species, and species name, abbreviation, and family are given. See also Table 1 and S1 Fig. * Not included in Fig 2 due to divergence in catalytic domain and incomplete PEF domain.
(PDF)

S1 Fig Evolutionary relationship of organisms used in the phylogeny. A brief schematic description of the evolutionary tree of the organisms used for the phylogeny.
(PDF)

S2 Fig Phylogeny of calpain families. Species abbreviations and their phylogenetic classification and common name are detailed in Table 1. All reference numbers for sequences are in S1 Table. The analysis is described in the methods (the plot is from the RAxML analysis). *Aplysia* calpains are in larger font and bolded as are bootstrap numbers referred to in the text that define families. Families are defined by the lines and the family name on the right.
(PDF)

S3 Fig CCal 1 autolysis is blocked by mutation of the catalytic cysteine to serine. (A) CCal 1b-FLAG (approximately 70ng/ul), with the catalytic cysteine intact or converted to serine, was incubated with or without 5mM CaCl_2_ for 1 hr. Thirty microliters of each reaction was subjected to SDS-PAGE, transferred to nitrocellulose membrane and probed with an antibody against the C-terminal FLAG tag. (B) Quantification of three independent experiments. A one-tailed T-test for independent samples of equal variance yielded p<0.05, represented by an asterisk (*). Error bars show SEM.
(PDF)
