# Supplementary material for: Novel calpain families and novel mechanisms for calpain regulation in Aplysia
Source: PLoS One. 2017 Oct 20;12(10):e0186646. doi: 10.1371/journal.pone.0186646 (PMC5650170; doi:10.1371/journal.pone.0186646)
Supplement: S1 Table — Accession numbers for all sequences used in the phylogenetic analysis. For sequences downloaded from transcriptome analysis the most recent download information is given. Sequences are arranged by species, and species name, abbreviation, and family are given. See also Table 1 and S1 Fig. * Not included in Fig 2 due to divergence in catalytic domain and incomplete PEF domain. (PDF) [file pone.0186646.s001.pdf]

S1 Table

Accession numbers for all sequences used in the phylogenetic analysis. For sequences downloaded from transcriptome analysis the most recent download information is given. Sequences are arranged by species, and species name, abbreviation, and family are given. See also Table 1 and Fig. S1.

* Not included in Figure 2 due to divergence in catalytic domain and incomplete PEF domain.

Amphimedon Queenslandica(AMP): Sponge Porifera,Prebilaterian

AMPCCAL	XP_003388253.1

Aplysia Californica(APL): Aplysia, Mollusk, Lophotrochozoa

APLATYP	XP_005089461.1
APLCCAL1	XP_012942076.1
APLCCAL2	XP_005093816.2
APLCCAL3	XP_012940412.1
APLCCAL4	XP_012942068.1
APLCCAL5	XP_012942074.1
APLCCAL6	XP_005105754.2
APLPALB	c125304_g3_i1 len=3129 aplysiagenetools.org
APLSOL	XP_012936257.1
APLTRA1	c103226_c0_seq3 length=4878 aplysiagenetools.org
APLTRA2	c120955_g1_i1 length=2580 aplysiagenetools.org
APLTRUNC1	XP_005105403.1
APLTRUNC2	c6_g1_i3 len=3371 aplysiagenetools.org
APLTRUNC3	XP_012935411.1

Branchiostoma Floridae(BRA): Lancelet, Tunicate, Chordate, Deuterostome

BRACCAL2	XP_002590877.1
BRACCAL1	XP_002590876.1
BRACCAL1B*	XP_002607981.1
BRASOL	XP_002611732.1
BRATRA	XP_002603439.1
BRATRUNC1	XP_002591816.1
BRATRUNC2	XP_002606475.1
BRAPALB	XP_002586006.1

Capitella Teleta (CAP): Polychaete Worm, Annelid, Lophotrochozoa

CAPATYP	ELU08108.1
CAPCCAL1	ELU02909.1
CAPCCAL2	ELU16041.1
CAPCCAL3	ELU01678.1
CAPCCAL4	ELU01103.1
CAPCCAL6	ELU16041.1
CAPPALB	ELU09786
CAPSOL	ELT93632.1
CAPTRA	ELU17460.1
CAPTRUNC	ELT92549.1
CAPTRUNC2	ELU07186.1

Capsaspora Owczarzaki (CAS): Capsapspora, Filasterea, Pre-Metazoan

CASPALB XP_004364591.2

Crassostrea Gigas (CRA): Oyster, Mollusk, Lophotrochozoa

CRAATYP XP_011414355.1
CRACCAL1 XP_011439866.1
CRACCAL2 XP_011420776.1
CRACCAL4 EKC39150.1
CRAPALB XP_011436287.1
CRATRA XP_019926655.1
CRATRA1 XP_011450582.1
CRATRUNC EKC29136.1
CRATRUNC2 EKC25048.1

Danio Rerio (DAN): Zebrafish, Chordate, Deuterostome

DANCCAL1 AAF82808.1
DANCCAL2 XP_009294661.1
DANCCAL3 XP_017207116.1
DANTRA5 NP_001073476.1
DANPALB7 NP_001128580
DANCCAL8 XP_009294657.1
DANCACL9 NP_001003501.1
DANATYP10 XP_698873.4
DANCCAL11 NP_956739.1
DANCCAL12 NP_001076532.2
DANCCAL13 XP_005170815.1
DANCCAL17 NP_998104.1
DANSOL XP_002663893.2

Daphnia Pulex (DAP): Water Flea, Crustacean, Ecdysozoa

DAPCCAL2 EFX86047.1
DAPCCAL1A EFX85068.1
DAPCCAL1B EFX75284.1
DAPPALB EFX79800.1

Drosophila Melanogaster (DRO): Fruit Fly, Insect, Ecdysozoa

DROCCALA NP_477047.1
DROCCALB NP_524016.4
DROCCALC NP_573118.2
DROSOL AEC46883.1

Fugu Rupripes (FUG): Pufferfish, Chordate, Deuterostome

FUGCCAL17 XP_011607025.1

Histoplasma (HIS): Fungi, Pre-Metazoan

HISPALB CAA91013.2

Homo Sapiens (HUM): Human, Chordate, Deuterostome

HUMCCAL1 EAW74368.1
HUMCCAL2 AAH07686.1

HUMCCAL3	EAW92550.1
HUMTRA5	NP_004046.2
HUMTRA6	NP_055104.2
HUMPALB7	EAW64224.1
HUMCCAL8	EAW93255.1
HUMCCAL9	EAW69922.1
HUMATYP10	AAG17971.1
HUMCCAL11	XP_006715050.1
HUMCCAL12	NP_653292.2
HUMCCAL13	XP_016860755.1
HUMCCAL14	NP_001138594.1
HUMSOL15	NP_005623.1

Limulus polyphemus (LIM): Horseshoe crab, Crustacean, Ecdysozoa

LIMTRA	XP_013788366.1
LIMTRUNC	XP_013789816.1

Lottia Gigantica (LOT): Limpet, Mollusk, Lophotrochozoa

LOTCAT1	XP_009065184.1
LOTCAT2	XP_009048751.1
LOTCCAL1	XP_009059203.1
LOTPALB	XP_009047414.1
LOTSOL	XP_009055297.1
LOTTRA1	XP_009044444.1
LOTTRA2	XP_009062876.1
LOTTRUNC1	XP_009051588.1
LOTTRUNC2	XP_009065346.1
LOTTRUNC3	XP_009066084.1

Mnenopsis Leidyi (MNE): Comb Jelly, Ctenophore, Pre-Bilaterian

MNETRUNC	ML022412a (Mnemiopsis leidyi prot2.2.aa.fa)
MNESOL	ML293116a (Mnemiopsis leidyi prot2.2.aa.fa)
MNEPALB	ML127014a (Mnemiopsis leidyi prot2.2.aa.fa)

Nematostella Vectensis (NEM): Sea Anemone, Cnidarian, Pre-Bilaterian

NEMCCAL2	XP_001640696.1
NEMCCAL1	XP_001632256.1
NEMPALB	XP_001637860.1
NEMSOL	XP_001630054.1
NEMTRA	XP_001626960.1

Strongylocentrotus Purpuratus(PUR): Sea Urchin,Echinoderm, Deuterostome

PURPALB	XP_011664595.1
PURCCAL1	XP_801749.2
PURSOL	XP_011669258.1
PURTRA	XP_001178522.1
PURTRUNC	XP_011660989.1

Rice: Plant (Outgroup)

RICEDEK	AAL38190.1

Salpingoeca Rosetta (SAL): Choanoflagellate, Pre-Metazoan

SALPALB	XP_004997995.1
SALSOL	XP_004995676.1

Trichoplax Adherens (TRI): Placazoa, Pre-Bilaterian

TRIPALB	XP_002111255.1
TRICCAL1	XP_002108316.1
TRITRA	XP_002107604.1
TRITRUNC	XP_002109313.1

Xenopus Tropicalis (XEN): Frog, Chordate, Deuterostome

XENCCAL1	NP_001080485.1
XENCCAL2	NP_001083713.1
XENCCAL3	XP_018083987.1
XENTRA5	NP_001080808.1
XENTRA6	XP_018085341.1
XENPALB7	XP_018122985.1
XENCCAL8	NP_0010820121
XENCCAL9	NP_001085997.1
XENATYP10	AAI08605.1
XENCCAL11	NP_001083150.1
XENCCAL12	XP_018085761.1
XENCCAL13	NP_001079488.1
XENCCAL14	XP_004914982.2
