# Supplementary figures and images for: Novel calpain families and novel mechanisms for calpain regulation in Aplysia
Source: PLoS One. 2017 Oct 20;12(10):e0186646. doi: 10.1371/journal.pone.0186646 (PMC5650170; doi:10.1371/journal.pone.0186646)

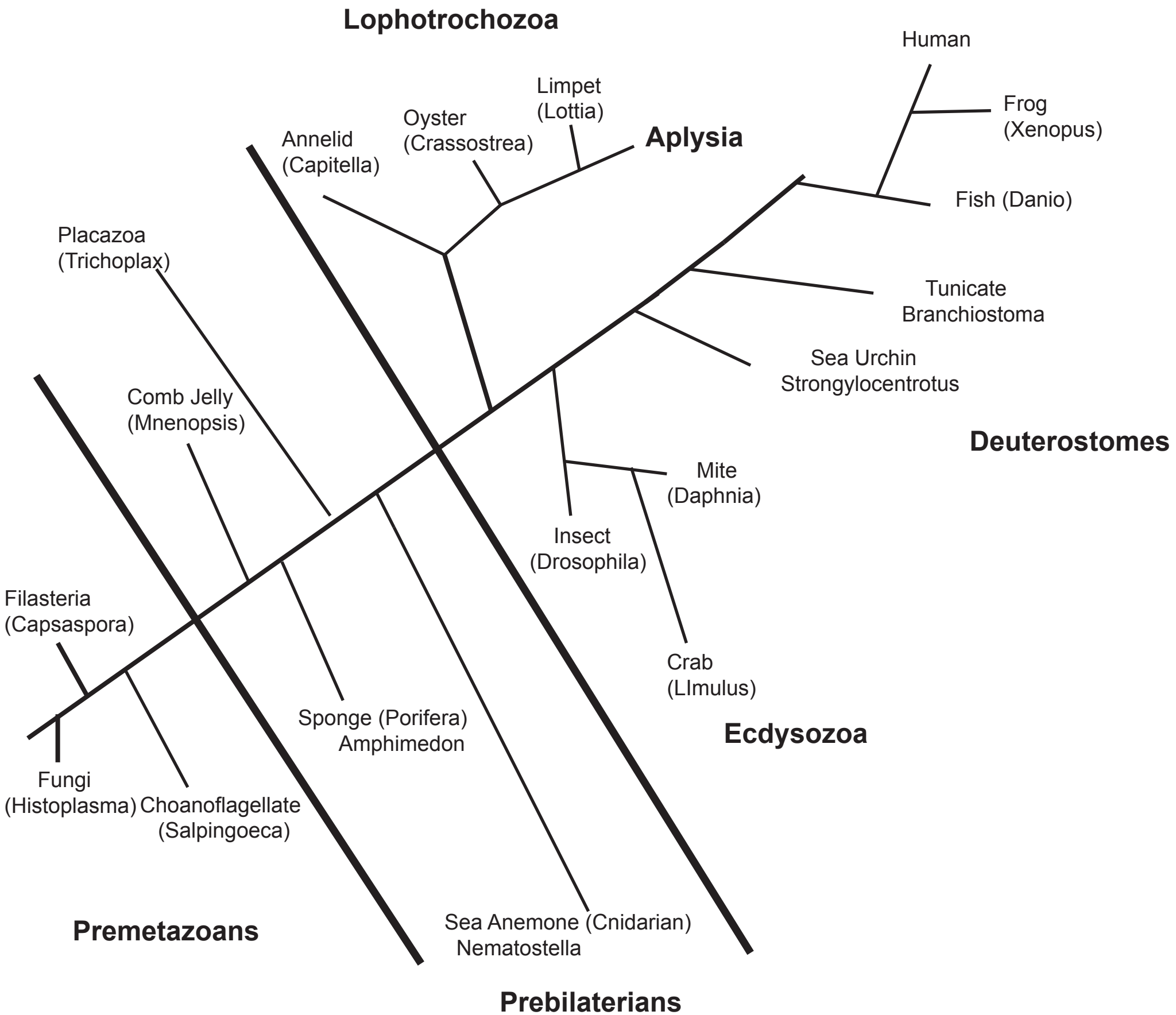

Supplement: S1 Fig — A brief schematic description of the evolutionary tree of the organisms used for the phylogeny. (PDF) [file pone.0186646.s002.pdf]

**Figure S3. CCal 1 autolysis is blocked by mutation of the catalytic cysteine to serin.**

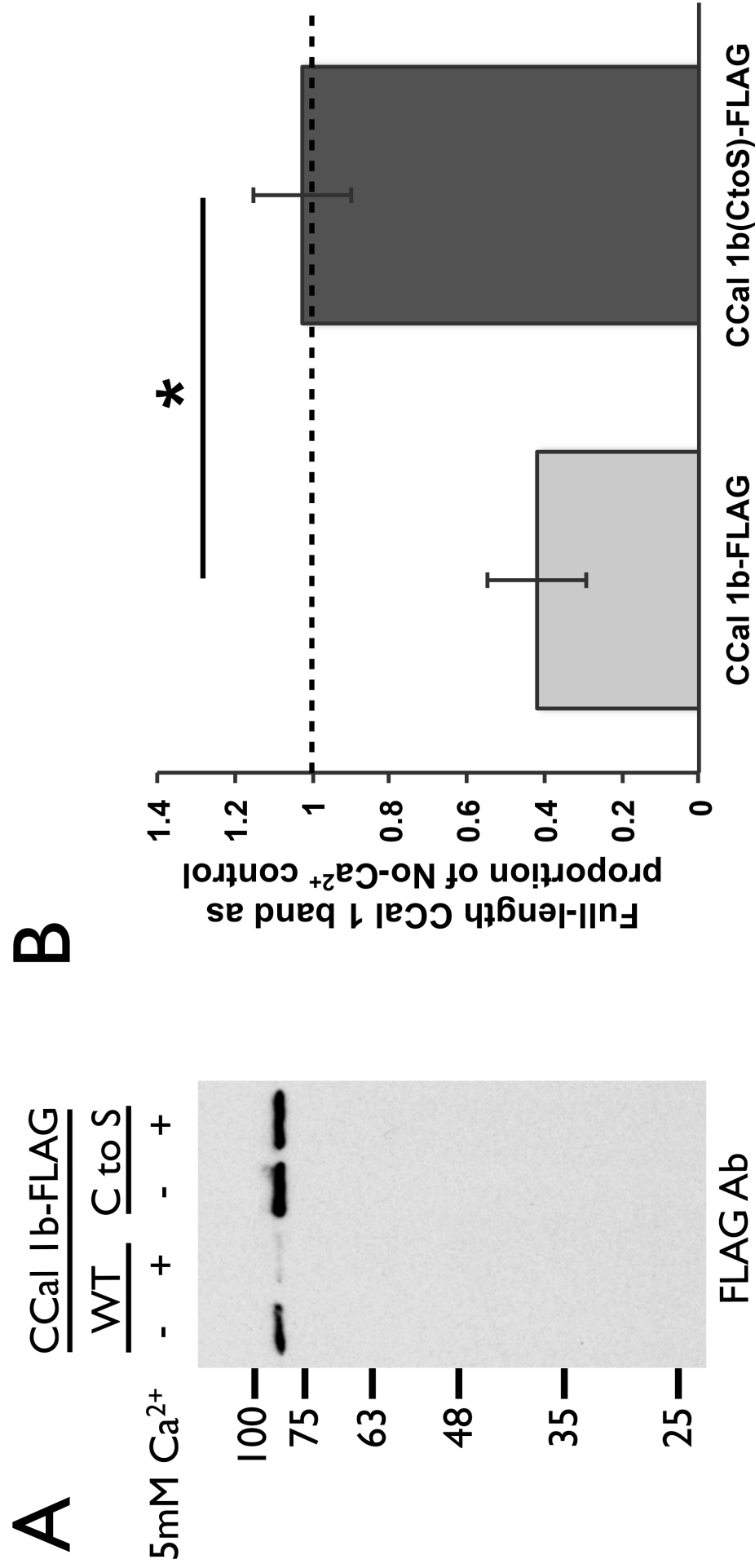

Supplement: S3 Fig — (A) CCal 1b-FLAG (approximately 70ng/ul), with the catalytic cysteine intact or converted to serine, was incubated with or without 5mM CaCl2 for 1 hr. Thirty microliters of each reaction was subjected to SDS-PAGE, transferred to nitrocellulose membrane and probed with an antibody against the C-terminal FLAG tag. (B) Quantification of three independent experiments. A one-tailed T-test for independent samples of equal variance yielded p<0.05, represented by an asterisk (*). Error bars show SEM. (PDF) [file pone.0186646.s004.pdf]
