# Supplementary material for: Novel calpain families and novel mechanisms for calpain regulation in Aplysia
Source: PLoS One. 2017 Oct 20;12(10):e0186646. doi: 10.1371/journal.pone.0186646 (PMC5650170; doi:10.1371/journal.pone.0186646)

Figure S2. Phylogeny of calpain families.
Species abbreviations and their phylogenetic classification and common name are detailed in Table 1. All reference numbers for sequences are in Table S1. The analysis is described in the methods (the plot is from the RAxML analysis). Aplysia calpains are in larger font and bolded as are bootstrap numbers referred to in the text that define families. Families are defined by the lines and the family name on the right.

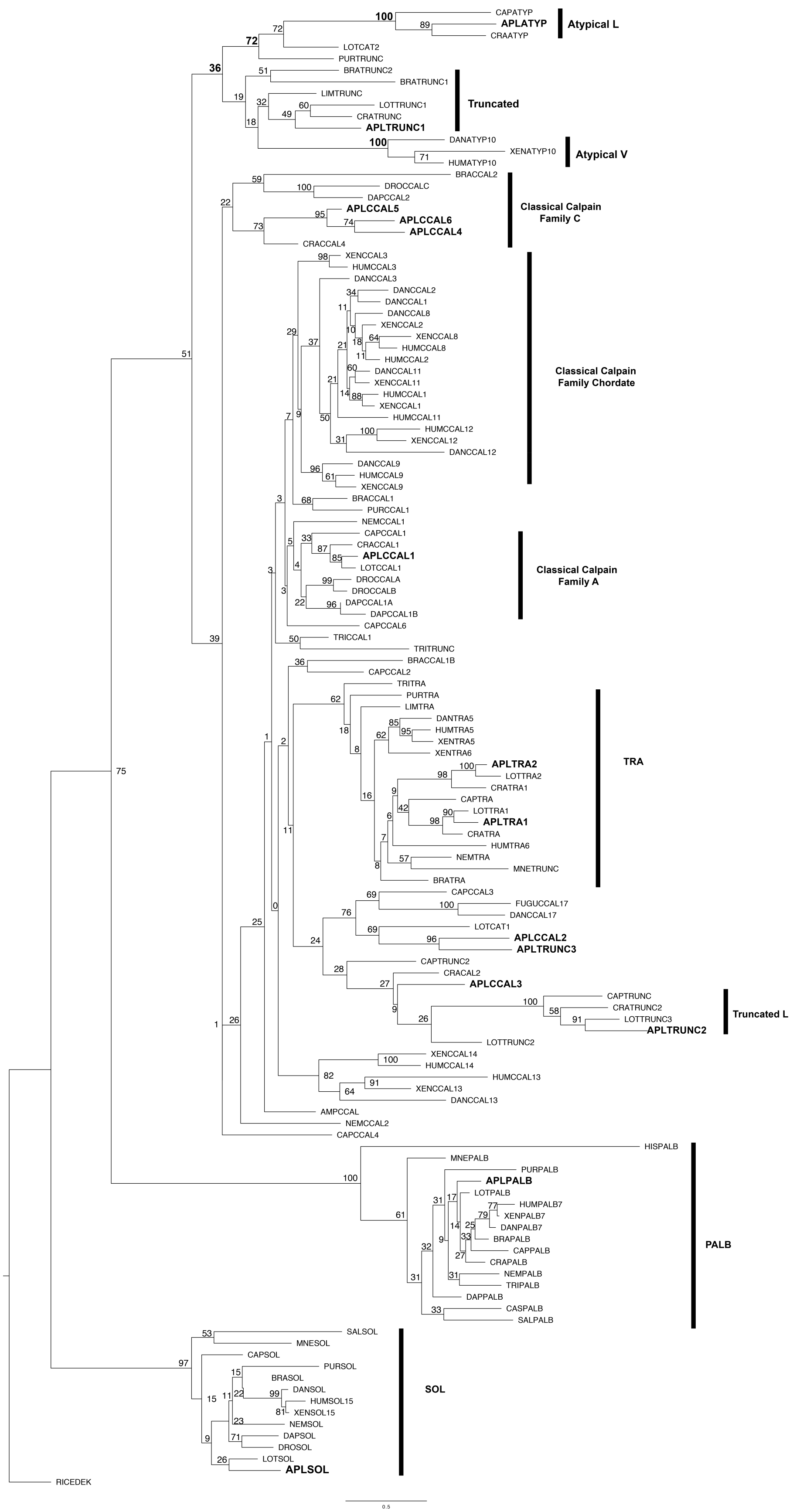

Supplement: S2 Fig — Species abbreviations and their phylogenetic classification and common name are detailed in Table 1. All reference numbers for sequences are in S1 Table. The analysis is described in the methods (the plot is from the RAxML analysis). Aplysia calpains are in larger font and bolded as are bootstrap numbers referred to in the text that define families. Families are defined by the lines and the family name on the right. (PDF) [file pone.0186646.s003.PDF]
